# Fast and selective fluoride ion conduction in sub-1-nanometer metal-organic framework channels

**DOI:** 10.1038/s41467-019-10420-9

**Published:** 2019-06-11

**Authors:** Xingya Li, Huacheng Zhang, Peiyao Wang, Jue Hou, Jun Lu, Christopher D. Easton, Xiwang Zhang, Matthew R. Hill, Aaron W. Thornton, Jefferson Zhe Liu, Benny D. Freeman, Anita J. Hill, Lei Jiang, Huanting Wang

**Affiliations:** 1 Department of Chemical Engineering, Monash University, Clayton, VIC 3800 Australia; 2 Department of Mechanical Engineering, The University of Melbourne, Parkville, VIC 3010 Australia; 3 Manufacturing, CSIRO, Clayton, VIC 3168 Australia; 4 Department of Chemical Engineering, The University of Texas at Austin, Austin, TX 78712 USA; 5 Key Laboratory of Bioinspired Materials and Interfacial Science, Technical Institute of Physics and Chemistry, Chinese Academy of Sciences, 100190 Beijing, People’s Republic of China

**Keywords:** Metal-organic frameworks, Organic-inorganic nanostructures

## Abstract

Biological fluoride ion channels are sub-1-nanometer protein pores with ultrahigh F^−^ conductivity and selectivity over other halogen ions. Developing synthetic F^−^ channels with biological-level selectivity is highly desirable for ion separations such as water defluoridation, but it remains a great challenge. Here we report synthetic F^−^ channels fabricated from zirconium-based metal-organic frameworks (MOFs), UiO-66-X (X = H, NH_2_, and N^+^(CH_3_)_3_). These MOFs are comprised of nanometer-sized cavities connected by sub-1-nanometer-sized windows and have specific F^−^ binding sites along the channels, sharing some features of biological F^−^ channels. UiO-66-X channels consistently show ultrahigh F^−^ conductivity up to ~10 S m^−1^, and ultrahigh F^−^/Cl^−^ selectivity, from ~13 to ~240. Molecular dynamics simulations reveal that the ultrahigh F^−^ conductivity and selectivity can be ascribed mainly to the high F^−^ concentration in the UiO-66 channels, arising from specific interactions between F^−^ ions and F^−^ binding sites in the MOF channels.

## Abstract (web summary)

While biological fluoride ion channels display excellent F^−^ conductivity and selectivity, designing synthetic analogues remains highly challenging. Here the authors show that zirconium-based metal–organic frameworks with F^−^ binding sites and sub-1-nanometer channels exhibit ultrahigh F^−^ conductivity and selectivity.

## Introduction

**Fig. 1:**
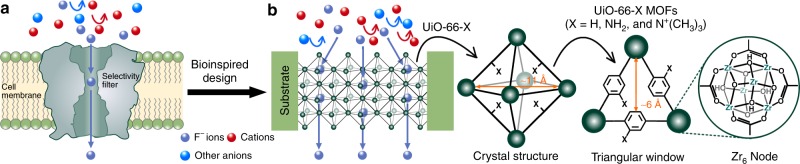
Bioinspired design of synthetic MOF channels for fluoride ion conduction. **a** Schematic of a biological fluoride (F^−^) ion channel with an angstrom-sized region as F^−^ selectivity filter and nanometer-sized vestibule and outlet for selective, ultrafast F^−^ transport. **b** Schematics of bioinspired artificial zirconium-based UiO-66-X (X = H, NH_2_, and N^+^(CH_3_)_3_) MOF channels with sub-1-nanometer crystalline pores for selective and ultrafast F^−^ transport. Sub-1-nanometer MOF channels consist of angstrom-sized triangular windows (~6 Å in diameter) for ion sieving and nanometer-sized octahedral cavities (~11 Å in diameter) for ultrafast ion conduction

Fluoride (F^−^) ions are ubiquitous in soil, groundwater and the ocean at levels of 10‒100 μM, posing a chronic threat to microorganisms^1^. Excessive (>1.5 mg L^−1^)^2^ and prolonged fluoride intake is highly detrimental to human health, leading to dental and skeletal fluorosis or neurological damage^3^. To lower the intracellular F^−^ concentration, fluoride ion channels have evolved in unicellular organisms to selectively transport F^−^, thereby reducing its concentration to alleviate toxicity^4,5^ (Fig. 1a). Despite the co-existence of F^−^ and other ions of the same valence and similar sizes, such as chloride (Cl^−^) ions, which are present at much higher concentration than F^−^ ions in biological systems^6^, the selectivity of F^−^ over Cl^−^ for most biological fluoride ion channels ranges from ~10 to 10,000^7^. The ultrahigh F^−^ selectivity presumably arises from angstrom-sized pores and anion-specific binding affinity with phenylalanine moieties in the F^−^ ion channels^7–10^. Inspired by natural F^−^ ion channels, construction of biomimetic fluoride ion channels within membranes is postulated to be an effective way to enhance the efficiency of F^−^ ion separation and, in turn, water defluoridation. While some efforts have been made to fabricate synthetic ion channels via anion-specific binding affinity, the F^−^/Cl^−^ selectivity of these channels is only around 1.8^11^, much lower than that of biological F^−^ ion channels. In addition, synthetic nanoporous polymer membranes have been developed to separate fluoride ions from other monovalent anions via electrostatic interaction or anion exchange, exhibiting F^−^/Cl^−^ selectivities of only ~1.4^12^ and ~0.8^13^, respectively. Compared with biological ion channels, the low selectivity of existing synthetic materials is due in part to their large nanopores, which do not provide a small enough confined space to guarantee that, when passing through the channel, each ion can effectively interact with any binding sites on the channel wall. Therefore, it remains a long-sought goal to develop artificial channels with angstrom-scaled dimensions similar to the size of halogen ions^14^.

Metal-organic frameworks (MOFs), an emerging family of porous crystalline materials, have high porosity, large surface area and uniform pore size, making them of great interest for gas storage^15,16^, capture^17,18^ and separation technologies^19–23^. In particular, water-stable MOFs that have angstrom-scaled pore windows have recently proven to be promising for water processing^24^. For instance, zeolitic imidazolate framework-8 (ZIF-8) contains 3.4 Å pore windows and has been theoretically predicted to be a potential reverse osmosis membrane material for water purification^25^. Subsequently, Huang et al. have experimentally demonstrated that ZIF-8 membranes can be used for seawater desalination via ionic sieving, achieving an ion rejection value of more than 99.8%^26^. Recently, zirconium based UiO-66 membranes with channels comprised of octahedral and tetrahedral cavities (~11 Å and ~9 Å, respectively) connected by ~6 Å pore windows^27,28^ have been fabricated by Li et al. These membranes achieve high rejection of multivalent metal ions that have hydrated ionic diameters (~7.0‒9.0 Å) larger than the MOF window size^29^. MOF particles with tailored functionalities have been shown to be efficient molecular and ionic adsorbents owing to specific interactions between the molecules/ions of interest and MOF active sites^30–33^. For example, arsenic ions have been removed from water by using UiO-66 crystals as adsorbents through the hydroxyl and benzenedicarboxylate binding sites^31^. Furthermore, a series of MOFs (i.e., MIL-53(Fe), MIL-53(Cr), CAU-6, UiO-66(Zr), UiO-66(Hf), ZIF-7, ZIF-8 and ZIF-9) has been explored as potential adsorbents for removing fluoride from water^34^. Based on these studies, UiO-66(Zr) derivative MOFs display the highest F^−^ adsorption capacity due to the specific binding of F^−^ through the hydroxyl and open zirconium sites^35,36^. Therefore, we hypothesize that UiO-66 MOFs having channels with angstrom-sized pore windows and specific F^−^ binding sites might be suitable candidates for constructing ion channels that offer highly selective and rapid transport of F^−^ ions.

Herein, we report ultrahigh fluoride ion conductive and selective MOF channels with sub-1-nanometer windows and a variety of functional groups constructed by in-situ growth of zirconium-based UiO-66-X (X = H, NH_2_, and N^+^(CH_3_)_3_) MOFs into polyethylene terephthalate (PET) nanochannels (Fig. 1b). The UiO-66-X MOF channels, which consist of angstrom-sized windows and nanometer-sized cavities with positive framework charges^37^, function as fluoride ion channels, exhibiting ultrahigh F^−^ conductivity and selectivity over other anions (F^−^ ≫ Cl^−^ > Br^−^ > I^−^ > NO_3_^−^ > SO_4_^2−^). The selectivity increases with increasing dehydrated anion diameter and can be tuned by varying the functional groups on MOFs. For instance, PET-UiO-66-N^+^(CH_3_)_3_ nanochannels have F^−^/Cl^−^ selectivity values up to ~240, rivaling those of many biological F^−^ ion channels^7,9^. Based on a combination of molecular dynamics simulations and experiments, we can attribute the high F^−^/Cl^−^ selectivity in UiO-66-X channels mainly to the high concentration of F^−^ ions in the sub-nanometer-sized MOF channels that arises from the strong binding between F^−^ ions and F^−^ specific binding sites in UiO-66-X frameworks.

## Results

### Fabrication of sub-1-nanometer porous MOF channels

**Fig. 2:**
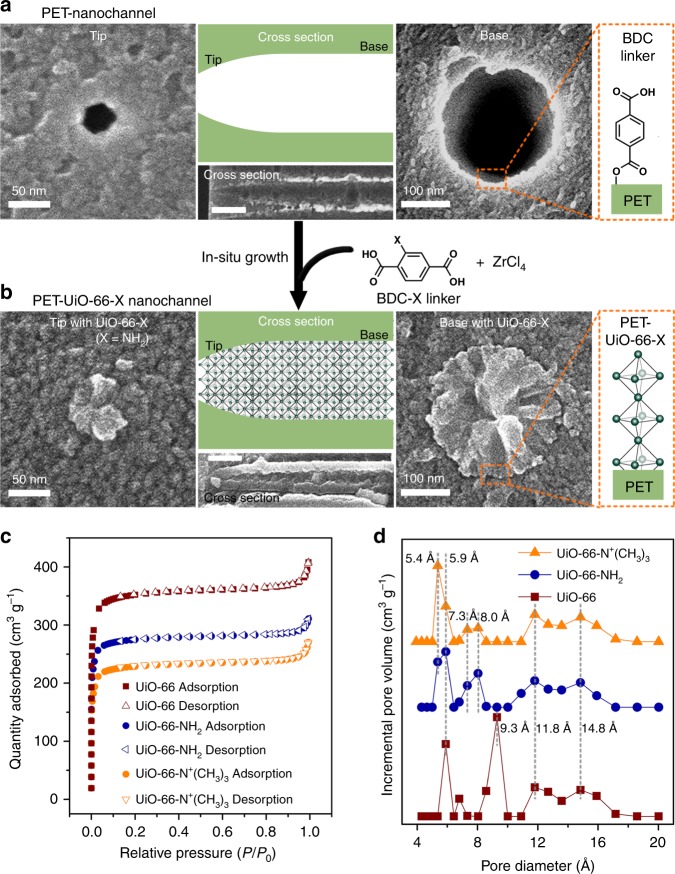
Fabrication and characterization of PET-UiO-66-X nanochannels. **a** Schematic of a single bullet-shaped nanochannel and SEM images of the channel tip, cross section (the scale bar is 500 nm) and base. Average tip diameter is 36.3 ± 5.6 nm, while average base diameter is 328.3 ± 35.2 nm. The PET nanochannel surface possesses BDC linkers for in-situ growth of UiO-66-X MOFs. **b** Schematic of a PET-UiO-66-X nanochannel and SEM images of the nanochannel tip, cross section (the scale bar is 500 nm) and base (SEM images of PET-UiO-66-NH_2_ are shown as an example). Schematic of UiO-66-X crystal structure along the PET-nanochannel surface. **c** N_2_ adsorption/desorption isotherms of UiO-66, UiO-66-NH_2_, and UiO-66-N^+^(CH_3_)_3_. **d** Pore size distributions of UiO-66-X MOFs. The distributions for UiO-66-NH_2_ and UiO-66-N^+^(CH_3_)_3_ are displayed vertically for ease of viewing. Pore size decreases as MOFs functional group size increases

Sub-1-nanometer MOF channels were fabricated by in-situ growth of UiO-66-X crystals into 12-µm-thick single-nanochannel PET membranes (see Methods for more details). Single bullet-shaped nanochannels embedded within a PET membrane (Fig. 2a) were fabricated by a surfactant-protected ion-track-etching method^38^. Scanning electron microscopy (SEM) images of the tip, tip cross section and base of the PET-nanochannel displayed a bullet-like nanochannel (Fig. 2a). The mean nanochannel tip diameter was 36.3 ± 5.6 nm, and the mean nanochannel base diameter was 328.3 ± 35.2 nm (Supplementary Fig. 1). Here, the bullet-shaped single-nanochannel PET membranes were prepared as supports to house MOFs in the nanochannels, since the asymmetric shape and the benzene-1,4-dicarboxylic acid (BDC)-linkers on the PET-nanochannel wall favor in-situ growth of UiO-66-X MOFs inside the nanochannels (Fig. 2a).

Following in-situ growth of UiO-66-X, the PET nanochannel was completely filled with UiO-66-X MOF crystals (Fig. 2b), which was verified by SEM images of the tip, tip cross section, and base of the PET-UiO-66-X nanochannel (Fig. 2b), as well as energy dispersive X-ray spectroscopy (EDX) mapping of the PET-nanochannel membrane before and after UiO-66-X growth (Supplementary Fig. 2a-d). The powder X-ray diffraction (XRD) patterns and X-ray photoelectron spectroscopy (XPS) spectra of PET and PET-UiO-66-X nanochannels confirmed the existence of UiO-66-X crystals in the PET nanochannels (Supplementary Fig. 2e, f). Based on N_2_ adsorption/desorption isotherm profiles, UiO-66-X crystals had Brunauer-Emmett-Teller (BET) surface areas of 1372 ± 8 m^2^ g^−1^, 1133 ± 11 m^2^ g^−1^ and 947 ± 13 m^2^ g^−1^ for UiO-66, UiO-66-NH_2_, and UiO-66-N^+^(CH_3_)_3_, respectively (Fig. 2c), calculated according to the four BET consistency criterion^39,40^. The pore size distributions revealed nanometer-sized cavities with ~6 Å window diameters and a gradual decrease in window size going from UiO-66, to UiO-66-NH_2_, and to UiO-66-N^+^(CH_3_)_3_, consistent with the increase in MOF functional group size (Fig. 2d). These results were further confirmed by the window and cavity sizes of UiO-66-X channels calculated via Zeo++ (see Supplementary Fig. 3 for more details).

### Ultrahigh F^−^ conductivity and selectivity in MOF channels

**Fig. 3:**
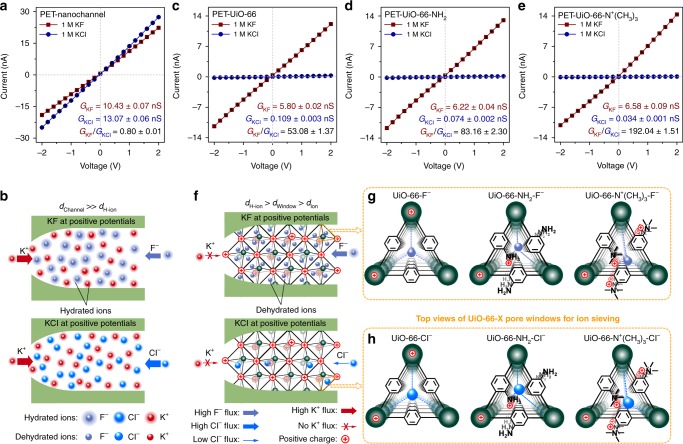
Ionic transport properties of PET nanochannels and sub-1-nanometer MOF channels. **a** Symmetric *I‒V* curves of a bullet-shaped PET-nanochannel observed in 1.0 M KF and KCl solutions, respectively. **b** Schematics of hydrated ions transport in a PET-nanochannel. Because *d*_Channel_ ≫ *d*_H-ion_, ions are hydrated in the PET-nanochannel. The diameter of hydrated F^−^ is larger than that of hydrated Cl^−^, so hydrated F^−^ ions conduct slower than hydrated Cl^−^ ions in the PET-nanochannel. **c**–**e** *I–V* curves of PET-UiO-66-X nanochannels measured in 1.0 M KF and KCl solutions, respectively. Compared with UiO-66 and UiO-66-NH_2_ channels, UiO-66-N^+^(CH_3_)_3_ channels exhibit the highest F^−^ conductance and selectivity. Error bars represent the standard deviation of three measurements of a sample. **f** Schematic illustrations of dehydrated ions passing through sub-1-nanometer PET-UiO-66-X nanochannels. Ions are dehydrated in MOF channels because *d*_H-ion_ *>* *d*_Window_ > *d*_Ion_. The angstrom-sized MOF pore windows with specific F^−^ binding zirconium sites and functional NH_2_ and N^+^(CH_3_)_3_ groups serve as F^−^ selectivity filter for ion sieving, so dehydrated F^−^ ions conduct faster than Cl^−^ in PET-UiO-66-X nanochannels. **g**, **h** Top view of UiO-66-X pore windows. UiO-66-N^+^(CH_3_)_3_ channels have the smallest window size and strongest F^−^ specific interactions due to the N^+^(CH_3_)_3_ groups compared with UiO-66 and UiO-66-NH_2_ channels

To investigate ion conduction in UiO-66-X filled PET nanochannels (PET-UiO-66-X), current-voltage (*I*–*V*) curves of a PET-nanochannel before and after UiO-66-X growth were measured in 1.0 M KF and KCl aqueous solutions (see Supplementary Fig. 4a, b for the testing apparatus). For pristine bullet-shaped PET-nanochannels (i.e., with no MOFs in the nanochannels), symmetric *I-V* curves were observed in both KCl and KF solutions (Fig. 3a). In the pristine PET nanochannels, the absolute values of KCl currents were slightly higher than those of KF, consistent with the slightly smaller diameter of hydrated Cl^−^ relative to F^−^ (i.e., F^−^ (7.04 Å) > Cl^−^ (6.64 Å)^41^, Supplementary Table 1). Since the two electrolyte solutions share the same cation (i.e., K^+^), differences in PET-nanochannel conductance are ascribed to the anions (i.e., F^−^ and Cl^−^). In the PET-nanochannel, because *d*_Channel_ (channel diameter) is much larger than *d*_H-ion_ (hydrated ionic diameter), ions should be hydrated, and both cations and anions pass through the PET-nanochannel (Fig. 3b). Based on PET-nanochannel conductance values (*G*) calculated from the slopes of the PET-nanochannel *I-V* curves, *G*_KF_ and *G*_KCl_ were 10.43 ± 0.07 nS and 13.07 ± 0.06 nS, respectively. That is, without MOFs, the PET nanochannels exhibit very limited anionic selectivity on the basis of the conductance ratio value (i.e., *G*_KF_/*G*_KCl_) of 0.80 ± 0.01.

After PET nanochannels were filled with UiO-66-X, absolute values of KF flux (i.e., current) at a given voltage were somewhat smaller than those in the pristine PET-nanochannel, but KCl current values were reduced by more than 100-fold at a given voltage relative to KCl current values in the pristine PET-nanochannel (Fig. 3c–e). *G*_KF_ values of PET-UiO-66, PET-UiO-66-NH_2_, and PET-UiO-66-N^+^(CH_3_)_3_ nanochannels were 5.80 ± 0.02 nS, 6.22 ± 0.04 nS, and 6.58 ± 0.09 nS, respectively. In sharp contrast, *G*_KCl_ values of the PET-UiO-66, PET-UiO-66-NH_2_, and PET-UiO-66-N^+^(CH_3_)_3_ nanochannels were only 0.109 ± 0.003, 0.074 ± 0.002, and 0.034 ± 0.001 nS, respectively. Consequently, the PET-MOF nanochannels exhibit ultrahigh *G*_KF_/*G*_KCl_ selectivity, varying from 53.08 ± 1.37 to 192.04 ± 1.51, which are much higher values than those of PET nanochannels (Supplementary Fig. 4c–f). The ultrahigh selectivity of these PET-MOF nanochannels is ascribed to the unique structure and chemical properties of sub-1-nanometer UiO-66-X channels.

PET-UiO-66-X nanochannels are positively charged in neutral water, and UiO-66-N^+^(CH_3_)_3_ has the highest positive charge density on MOF crystals, as indicated by zeta potential in Supplementary Table 2. UiO-66 and UiO-66-NH_2_ are positively charged because the Zr–OH on the Zr_6_ node tends to form Zr–OH_2_^+^ in aqueous solution when the pH value is below 8.3^42^, while the amino group of UiO-66-NH_2_ can be protonated when the solution pH value is below 5, and UiO-66-N^+^(CH_3_)_3_ is positively charged mainly because of its quaternary ammonium groups (cf. Supplementary Fig. 5). Accordingly, the highly positively charged sub-1-nanometer MOF channels preferentially transport anions via electrostatic attraction and exclude cations (e.g., K^+^) via electrostatic repulsion, and XPS spectra of KF- and KCl-treated UiO-66-X crystals showed that F^−^ concentrations were much higher than Cl^−^ concentrations in UiO-66-X channels because additional F^−^ ions can be adsorbed into UiO-66-X channels by Zr sites through specific Zr–F interactions, while Cl^−^ ions cannot (Supplementary Tables 3–5 and Supplementary Fig. 6a–d). The exclusion of cations was confirmed by XPS spectra of KF- and KCl-treated UiO-66-X crystals. No detectable peaks associated with K 2 p or K 2 s were observed in the XPS spectra at low ion concentrations (<0.5 M), while K 2p peaks were observed in the XPS spectra at high KF concentrations (≥0.5 M) (Supplementary Tables 3–5). To determine the concentration of K^+^ ions in MOF channels at low external ion concentrations, we used inductively coupled plasma optical emission spectrometry to measure the ion concentration changes of bulk 0.01 M KCl and KF solutions before and after exposure to UiO-66 crystals. 0.1 g UiO-66 crystals adsorbed ~0.192 mmol F^−^ ions and ~0.019 mmol K^+^ from 0.01 M KF solution, and the ratio of F^−^/K^+^ in UiO-66 channel was ~10 (Supplementary Fig. 6e). In contrast, a negligible K^+^ adsorption capacity of ~0.016 mmol g^−1^ was observed in 0.01 M KCl solution (Supplementary Fig. 6f). These results unambiguously confirmed that only a small amount of K^+^ ions was present in the UiO-66-X MOF channels. Therefore, the ion conductance of PET-MOF nanochannels is derived mainly from the movement of anions ($G \approx G_ -$, i.e., $G_{{KF}} \approx G_{{F}^ - }$), while the ion conductance of a pristine PET-nanochannel, containing no MOFs, is due to the movement of both cations and anions ($G = G_ + + G_ -$, i.e., $G_{{KF}} = G_{{K}^ + } + G_{{F}^ - }$).

To further demonstrate the anion selectivity of PET-MOF nanochannels, anion conductivities (*κ*) of PET and PET-MOF nanochannels were calculated (see Methods for details). Experimental *κ*_*KF*_ and *κ*_*KCl*_ values for 1.0 M bulk solutions were 8.531 S m^−1^ and 10.820 S m^−1^, respectively. Pristine PET-nanochannels exhibited conductivities similar to those in bulk solution (Supplementary Fig. 7a). For bulk solutions, $\kappa = 10^3(\mu _ + + \mu _ - ) \times c \times F$, where *μ* is the ion mobility^43^ (Supplementary Table 1), *c* is the salt concentration, and *F* is Faraday’s constant. Based upon this formula, the anion conductivity in bulk solution can be calculated as $\kappa _ - = \kappa _{E{\mathrm{xp}}} \,\mu _ - /(\mu _ + + \mu _ - )$, where *κ*_*Exp*_ is the experimental conductivity value of the electrolyte solution. Accordingly, 1.0 M bulk $\kappa _{F^ - }$and $\kappa _{{\mathrm{Cl}}^ - }$ values were calculated to be 3.650 S m^−1^ and 5.511 S m^−1^, respectively. Similarly, $\kappa _{{\mathrm{F}}^ - }$and $\kappa _{{\mathrm{Cl}}^ - }$ values of pristine PET-nanochannels were 3.810 ± 0.025 S and 5.680 ± 0.027 S m^−1^, respectively. For PET-MOF channels, based on the assumption that all conductivity is attributed to the anion, $\kappa _ - \approx \kappa _{{\mathrm{Exp}}}$. $\kappa _{{\mathrm{F}}^ - }$of UiO-66, UiO-66-NH_2_, and UiO-66-N^+^(CH_3_)_3_ filled PET nanochannels were 6.969 ± 0.030, 9.169 ± 0.060, and 10.420 ± 0.142 S m^−1^, respectively. Interestingly, these values are considerably higher than the fluoride conductivity in bulk solution (i.e., 3.650 S m^−1^). Values of $\kappa _{{\mathrm{Cl}}^ - }$ for UiO-66, UiO-66-NH_2_, and UiO-66-N^+^(CH_3_)_3_ filled PET nanochannels were 0.131 ± 0.004, 0.110 ± 0.004, and 0.054 ± 0.001 S m^−1^, respectively (Supplementary Fig. 7b). These values are far below the chloride conductivity in bulk solution (5.511 S m^−1^). Based on these results, PET-UiO-66-X nanochannels exhibit much higher F^−^ conductivity than that measured in bulk solution or a pristine PET-nanochannel, and PET-UiO-66-N^+^(CH_3_)_3_ nanochannels have the highest F^−^ conductivity and F^−^/Cl^−^ selectivity among the MOFs considered.

The pore windows of UiO-66-X crystals are approximately 6.0 Å in diameter (Fig. 2d). The diameters of the hydrated anions considered in this study are all greater than 6.0 Å (Supplementary Table 1). However, the diameters of the dehydrated anions are less than 6.0 Å (Supplementary Table 1). That is, *d*_H-ion_ > *d*_Window_ > *d*_Ion_, where *d*_Ion_ is the dehydrated ion diameter, so any anions transported through MOF-filled PET nanochannels must be at least partially dehydrated to permeate through the pore windows of PET-UiO-66-X nanochannels (Fig. 3f–h). In addition to the dehydration effect, the sub-1-nanometer MOF channels possess a specific binding affinity for F^−^ over Cl^−^ due to the zirconium sites on the Zr-nodes and positively charged amino and quaternary ammonium groups^34,35^ (Supplementary Fig. 6a–c). This selective affinity also contributes to the ultraselective F^−^ transport in MOF channels. Moreover, the F^−^ conductivity increases slightly, while Cl^−^ conductivity decreases noticeably with an increase in the size of functional group in MOF channels (i.e., from UiO-66, to UiO-66-NH_2_, and to UiO-66-N^+^(CH_3_)_3_), resulting in the increasing of F^−^/Cl^−^ selectivity (Fig. 3c–e). Going from UiO-66, to UiO-66-NH_2_, and to UiO-66-N^+^(CH_3_)_3_ MOF is accompanied by a gradual decrease in window size, and the smaller the aperture size, the lower is the conductivity of anions, such as Cl^−^, through the MOF channels. In addition, interactions between functional MOFs and F^−^ ions may influence the selectivity. For example, quaternary UiO-66-NH_2_ (UiO-66-N^+^(CH_3_)_3_), which has the most positive charge density, exhibits stronger electrostatic interaction with F^−^ than UiO-66-NH_2_ and UiO-66 do. These interactions lead to more F^−^ ions entering the UiO-66-N^+^(CH_3_)_3_ channels (Supplementary Fig. 6a–c), a conclusion deduced from the zeta potential values of UiO-66-X after being loaded with F^−^ ions, where UiO-66-N^+^(CH_3_)_3_ has the most negative zeta potential value (Supplementary Table 2). Consequently, the PET-UiO-66-N^+^(CH_3_)_3_ nanochannels exhibit the highest F^−^/Cl^−^ selectivity, upto ~192, making them even more selective than most biological and synthetic fluoride ion channels (Supplementary Tables 6, 7). Therefore, specific interactions between F^−^ ions and the functional groups/sites on MOF frameworks may well be essential for the observed ultraselectivity of PET-MOF nanochannels.

### F^−^/Cl^−^ selectivity mechanisms in MOF channels

**Fig. 4:**
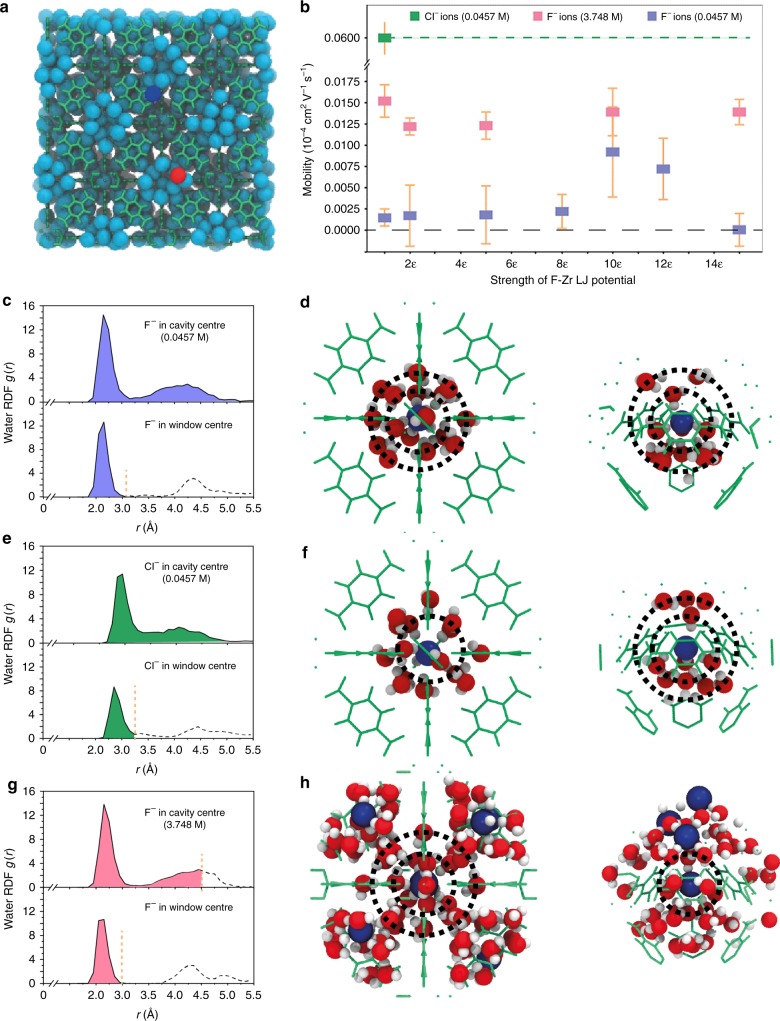
MD simulations of ion transport in UiO-66 channels. **a** The simulation cartoon shows UiO-66 cavities filled with water molecules (sky blue spheres), and they are connected via narrow windows. For clarity, UiO-66 is shown as a green wireframe. K^+^ and F^−^ ions are represented by red and dark blue spheres, respectively. **b** The mobility of Cl^−^ and F^−^ ions in UiO-66. F^−^ mobility is presented as a function of the strength of F–Zr LJ potential at 0.0457 M and 3.748 M, respectively. At 3.748 M, F^−^ mobility is enhanced by around 10 times compared with that at 0.0457 M. Error bars represent the standard deviation of calculations of 5 samples. **c, e, g** Radial distribution function of water molecules around anions sitting at cavity center and window center. Cl^−^ ions have a weaker second hydration shell compared with F^−^ ions at 0.0457 M when sitting at cavity center (c, e). However, at 3.748 M, F^−^ ions have a relative smaller hydration shell as part of water molecules within second shell were shared with neighbored F^−^ ions (g). **d, f, h** The simulation cartoon shows the arrangement of water molecules around anions sitting at cavity center (left) and window center (right), corresponding to c, e and g, respectively. Water molecules are red (O) and white (H), and ions (F^−^ or Cl^−^) are dark blue. F^−^ ions have two hydration layers at cavity center, and the second hydration shell should peel off at window center at 0.0457 M (d). Cl^−^ ions do not have a distinct second hydration shell (f), thus, smaller dehydration energy would be required for transport through windows compared with F^−^ ions at 0.0457 M. At 3.748 M, water molecules of F^−^ second hydration shell are shared with neighbored F^−^ ions (h)

To gain a deeper understanding of the observed ultrahigh F^−^/Cl^−^ selectivity in UiO-66-X channels, we performed molecular dynamics (MD) simulations. The ions transport of KF and KCl in UiO-66 channels under an external electric field was simulated as an example. Figure 4a depicts the molecular system of our MD simulations (See more details in Methods).

The ion conductivity in MOF channels depends on ion concentration and mobility. We initially investigated F^−^ and Cl^−^ ion mobility in UiO-66 channels to determine whether ions might exhibit anomalous transport behavior in sub-nanometer-sized UiO-66, owing to the sub-nano-confinement effect and specific ion-surface interactions^44^. To simulate a dilute concentration of Cl^**−**^ in UiO-66 in our experiments, one pair of K^+^ and Cl^−^ ions was placed in a 2 × 2 × 2 supercell, corresponding to a concentration of 0.0457 M. For comparison, similar MD simulations were performed for F^−^ ions. Figure 4b and Supplementary Table 8 show mobility results. The Cl^−^ ion has a mobility of 0.058 ± 0.013 × 10^−4^ cm^2^ V^−1^ s^−1^, whereas the F^−^ ion has a much lower mobility, 0.0015 ± 0.001 × 10^−4^ cm^2^ V^−1^ s^−1^. These results are reasonably close to the mobilities estimated by using the umbrella sampling method and transition state theory^45,46^ (see details in Methods, Supplementary Fig. 8, and Supplementary Fig. 9). Figure 4c–e depict the radial distribution function (RDF), *g*(*r*), of water molecules around the anions in the center of the large octahedral cavity and the pore window, respectively. Compared with F^−^, the second hydration shell of Cl^−^ appears much weaker, which is confirmed in the molecular cartoons in Fig. 4d–f. The cartoons also indicate that the second hydration shell is removed for anions passing through the window (*i.e*., the sieving mechanism). Owing to the tightly bonded second shell in the octahedral cavity (Fig. 4d), a higher dehydration energy should be required for F^−^ ions to transport through the small windows, leading to much smaller mobility for F^−^ than for Cl^−^ (Fig. 4b). However, this result is precisely opposite to the observed high F^−^ conductivity in our experiments, confirming the indispensable role of ion concentration on the measured high F^−^/Cl^−^ selectivity.

**Fig. 5:**
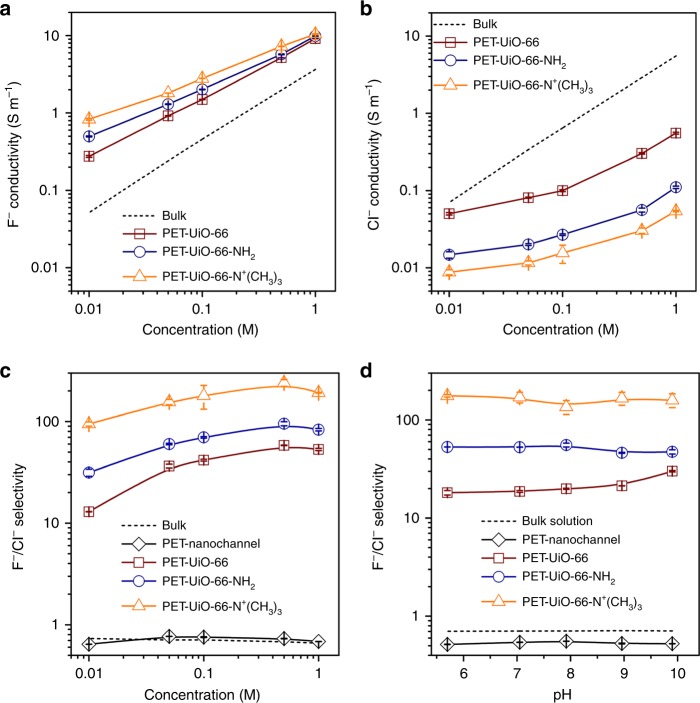
Influence of concentration and pH on F^−^, Cl^−^ conductivity and F^−^/Cl^−^ selectivity. **a**, **b** F^−^ and Cl^−^ conductivities in PET-UiO-66-X nanochannels as a function of ion concentration. F^−^ conductivities in UiO-66-X nanochannels are much higher than those in bulk solution, while Cl^−^ conductivities in UiO-66-X nanochannels are much lower than those in bulk solution. **c** F^−^/Cl^−^ selectivity of PET-UiO-66-X nanochannels as a function of ion concentration. Among the MOFs considered, the UiO-66-N^+^(CH_3_)_3_ nanochannel exhibits the highest F^−^/Cl^−^ selectivity (~240). **d** F^−^/Cl^−^ selectivity of PET-UiO-66-X nanochannels measured in 0.1 M KF and KCl solutions at different pH values. Error bars represent the standard deviation of three measurements of a sample

XPS results suggest that the F^−^ ion has strong interactions with Zr sites in UiO-66 channels (Supplementary Fig. 6d and Supplementary Tables 3, 4), and thus a much higher concentration (~100 times, Supplementary Table 4) than Cl^−^. To model the strong F–Zr binding, we manually increased the magnitude of parameter epsilon (*ε*) in the Lennard-Jones potential that characterizes the van der Waals (vdW) interaction strength between F^−^ and Zr sites^47^. To estimate the thermodynamic equilibrium F^−^ concentration in UiO-66 channels at the increased *ε* values, we adopted a relatively simple MD simulation model (Supplementary Fig. 10). Our model includes a UiO-66 slab (with several half cavities exposed at surfaces) connected to two electrolyte reservoirs having a constant electrolyte ion concentration of 1.0 M. After thermodynamic equilibration in MD simulations, the ion/water number ratio in the half cavities was used to estimate the ion concentration in UiO-66. At 15*ε*, the F^−^ ion concentration is approximately 3.6 M. Our MD simulations (details in Methods) show that placing 96 F^−^ ions in the UiO-66 supercells leads to equilibrium concentration of 3.748 M (i.e., ~3.6 M). This concentration is about 82 times higher than the dilute case (0.0457 M) in our MD simulations, very near the concentration difference measured in experiments (Supplementary Table 4). In these supercells, the F^−^ mobility is calculated as 0.0139 ± 0.0015 (unit: 10^−4^ cm^2^ V^−1^∙s^−1^), which is about one quarter of the Cl^−^ ion mobility at 0.0457 M. The ion conductivity ratio of F^−^/Cl^−^ is thus (3.748 × 0.0139 × 10^−4^)/(0.0457 × 0.058 × 10^−4^) = ~20, which lies in the range (10–50) of experimental observations of PET-UiO-66 channels presented in Fig. 5c. Our experiments and MD simulations results indicate that the observed high F^−^/Cl^−^ selectivity derives from the high F^−^ concentration in the UiO-66 channels, which, in turn, arises from the strong binding between F^−^ ions and Zr sites in UiO-66 channels, which is analogous to the natural fluoride ion channels.

Interestingly, our MD simulations show a much higher (~10 times) F^−^ ion mobility in the UiO-66 channels at an external electrolyte concentration of 3.748 M relative to that at 0.0457 M (Fig. 4b). This result is opposite to the bulk electrolyte result. Considering that F^−^ ion transport in UiO-66 channels is an activated hopping process between cavities (Fig. 4c, d), the ion concentration (which affects ion hydration in the cavities) and specific ion-channel interactions (mainly through F–Zr) should be the two primary factors influencing ion mobility. To examine their effects, we first reduced the F–Zr interaction strength from 15*ε* to 1*ε*, while keeping the F^−^ concentration at 3.748 M. The small mobility changes in Fig. 4b as a result of this change suggest that the ion concentration plays a dominant role. Analysis of our MD simulations shows that all ions were mobile (Supplementary Fig. 11). Stronger F–Zr interactions do not necessarily lead to immobilization of ions. Next, we reduced the F^−^ concentration to 0.0457 M, where F^−^ mobility is always lower, and varied the interaction strength, again resulting in small mobility changes (Fig. 4b). Our MD results, therefore, indicate that although the strong ion-channel (F–Zr) binding plays a direct role in ion adsorption concentration (thermodynamics), its direct effect on mobility (kinetics) is minor.

To gain further molecular level insights, the water RDFs surrounding F^−^ ions were calculated at the centers of the nanometer-sized cavities and sub-1-nanometer-sized windows for F^−^ concentration of 3.748 M (Fig. 4g). A comparison with Fig. 4c does not reveal significant differences. But careful inspection of the molecular cartoons in Fig. 4h shows that some water molecules in the second hydration shell are shared with neighboring F^−^ ions. This tendency may also be reflected by the slight increase in the second hydration shell position at high concentration. To illustrate the shared second hydration shell in *g(r)*, we applied a cut-off in Fig. 4g. The cut-off radius values were selected according to the averaged distances among F^−^ ions, which were determined from the radial distribution function of F^−^ ions around a F^−^ ion fixed in the large cavity center and window center of UiO-66 (Supplementary Fig. 12). For F^−^ sitting at the window center, there is only a minor change in *g*(*r*), and the molecular cartoon also shows the first hydration shell is largely to be intact. Our results suggest that the weaker second hydration shell (or partially dehydrated anion) at high concentration might be the reason for the enhanced mobility.

### Ion concentration and pH effects on F^−^/Cl^−^ selectivity

The dependence of ionic conductivity on ion concentrations in the aqueous solutions contiguous to the nanochannels was studied by varying KF and KCl concentrations from 0.01 to 1.0 M (see Supplementary Fig. 13 for *I-V* curves at different external electrolyte solution concentrations). In the pristine PET-nanochannel, the chloride ion conductivity is slightly higher than that of fluoride at the same concentration, consistent with the slightly smaller diameter of hydrated chloride (6.64 Å) relative to hydrated fluoride (7.04 Å) (Supplementary Fig. 14 and Supplementary Table 1). For UiO-66-X filled PET nanochannels, the F^−^ conductivity is much higher than that of Cl^−^ at the same voltage and concentration. In addition, both F^−^ and Cl^−^ conductivities increase with increasing ion concentration, but F^−^ conductivity rises more dramatically than that of Cl^−^ (Fig. 5a, b). Since PET-UiO-66-X nanochannels are F^−^ selective, the higher the concentration of F^−^ in the contiguous solutions, the more F^−^ ions can enter the MOF channels, which results in a higher F^−^ concentration, as well as a higher F^−^ mobility in the nanochannels, according to MD simulations. The F^−^/Cl^−^ selectivity of the pristine PET-nanochannel remains quite similar to that of bulk solution and is below 1 (Fig. 5c). In marked contrast, the F^−^/Cl^−^ selectivities for UiO-66, UiO-66-NH_2_ and UiO-66-N^+^(CH_3_)_3_ filled PET nanochannels increase with the decreasing of MOF pore sizes, and the F^−^/Cl^−^ selectivity of the PET-UiO-66-N^+^(CH_3_)_3_ nanochannel increased from ~95 to ~240 as external salt concentration rose from 0.01 to 0.5 M (Fig. 5c).

Since the Zr-nodes and amino groups on these MOFs are sensitive to pH, we measured conductivities of PET-UiO-66-X nanochannels in 0.1 M electrolyte solutions while varying pH from 5.7 to 10. Relative to the nearly constant conductivities of bulk electrolyte solutions at different pH values (Supplementary Fig. 15a), ionic conductivities of the pristine PET-nanochannel increase with the increasing electrolyte solution pH (Supplementary Fig. 15b). The p*K*_a_ of the COOH group of PET is ~3.8; as pH increases from 5.7 to 10, the PET-nanochannel becomes more negatively charged, since more COOH groups are deprotonated, thus attracting more cations to transport through the channel. However, as for UiO-66, the p*K*_a_ values of the *µ*_3_–OH, Zr–OH_2_, and Zr–OH groups on the Zr-nodes are about 3.52, 6.79, and 8.30, respectively; for UiO-66-NH_2_, the p*K*_a_ of the NH_2_ group on the ligand is about 5^48-50^; for UiO-66-N^+^(CH_3_)_3_, the framework is positively charged due to the quaternary ammonium groups (Supplementary Fig. 5). As pH in KF or KCl solution increases from 5.7 to 10, the degree of protonation degrees of the three MOFs vary insignificantly, as indicated by the zeta potential values shown in Supplementary Table 9. Consequently, F^−^ and Cl^−^ conductivities of the PET-UiO-66-X nanochannels are independent of pH over the range from 5.7 to 10 (Supplementary Fig. 15c–e). The F^−^/Cl^−^ selectivity of PET-UiO-66-X nanochannels varies slightly as pH increases from 5.7 to 10, and the selectivity values are extraordinarily higher than those of the pristine PET-nanochannel or bulk solution, which have a selectivity of less than 1. (Fig. 5d).

### Fluoride ion selectivity over other anions in MOF channels

**Fig. 6:**
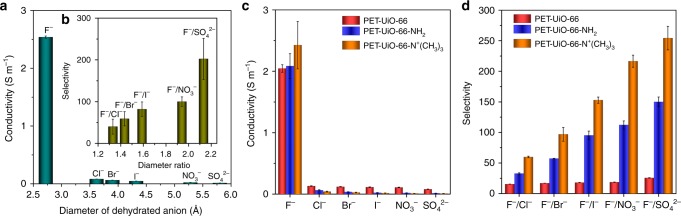
F^−^ selective properties of PET-UiO-66-X nanochannels. **a** Ionic conductivities of a PET-UiO-66-NH_2_ nanochannel decrease as dehydrated anion diameters increase. **b** Average ion selectivity as a function of dehydrated anion diameter ratio (Selectivity = $k_{{\mathrm{F}}^{-}}$/*k*_Anion_, diameter ratio = *d*_Anion_/$d_{{\mathrm{F}}^{-}}$). Error bars represent the standard deviation of independent measurement of three samples. **c**, **d** Systematic comparison of ionic conductivities and selectivities of PET-UiO-66, PET-UiO-66-NH_2_, and PET-UiO-66-N^+^(CH_3_)_3_ nanochannels. The PET-UiO-66-N^+^(CH_3_)_3_ nanochannel exhibits the highest F^−^ conductivity and selectivity. All data were obtained using 0.1  M electrolyte solutions at a pH value of 5.7. Error bars represent the standard deviation of three measurements of a sample

The selectivity of F^−^ over other anions in the PET-UiO-66-NH_2_ nanochannel was investigated by measuring the ionic currents with various electrolyte solutions including KCl, KBr, KI, KNO_3_, K_2_SO_4_, and KF (0.1 M, pH 5.7). As discussed earlier, the UiO-66-NH_2_ framework is positively charged in solution due to the Zr-sites present on the framework. Consequently, smaller anions can conduct faster through UiO-66-NH_2_ channels, while cations (i.e., K^+^) are blocked. From the *I-V* curves of a PET-UiO-66-NH_2_ nanochannel, the absolute ionic current of F^−^ at any voltage is much higher than that of other anions under the same conditions (Supplementary Fig. 16a, b), and their ionic current values decrease as dehydrated ionic diameter increases (Supplementary Table 1). In bulk solution and a pristine PET-nanochannel, conductivities of the hydrated monovalent anions decrease with increasing hydrated anionic diameters (Supplementary Table 1), while the divalent anion SO_4_^2−^ (hydrated ionic diameter of 7.58 Å) exhibits a higher conductivity than other hydrated monovalent anions (Supplementary Fig. 16c, d and Supplementary Table 1). In PET-UiO-66-NH_2_ nanochannels, however, anions conductivities decrease as dehydrated ion diameters increase, and F^−^ ions conduct much faster than other anions, owning to the specific binding effect discussed earlier (Fig. 6a). The average anion selectivity increased from a F^−^/Cl^−^ selectivity of 40.2 ± 17.9 to a F^−^/SO_4_^2−^ of 202.4 ± 49.2 as dehydrated ionic diameters increase (Fig. 6b; see Supplementary Table 10 for individual test results), which is much higher than that measured in a pristine PET-nanochannel or bulk solution (Supplementary Fig. 16e). The UiO-66 and UiO-66-N^+^(CH_3_)_3_ nanochannels display conductivity and selectivity similar to those of the UiO-66-NH_2_ nanochannel (Fig. 6c, d).

### Cycle performance and stability of MOF channels

To confirm the cyclability of PET-UiO-66-X nanochannels, ion current values of KF, KCl, KBr, and KI solutions (0.1 M, pH 5.7) were cyclically tested. After testing for at least three cycles, no obvious attenuation in current was observed in PET-UiO-66-NH_2_ nanochannels (Supplementary Fig. 17). XRD patterns of UiO-66-X crystals before and after treatment in KF, KCl, KBr, and KI solutions for 3 days demonstrated the stability of UiO-66-X MOFs (Supplementary Fig. 18). Furthermore, the BET surface areas and pore size distributions of UiO-66-NH_2_ crystals show only minor changes before and after treatment in KF, KCl, KBr, and KI solutions for 3 days (Supplementary Fig. 19).

## Discussion

In summary, highly conductive and selective fluoride ion channels have been constructed by in-situ growth of Zr-based UiO-66 derivative MOFs into asymmetric single-nanochannel PET membranes. The PET-UiO-66-X nanochannels exhibit remarkably high selectivity for F^−^ over Cl^−^, Br^−^, I^−^, NO_3_^−^, and SO_4_^2−^. Our experiments and MD simulations indicate that the high F^−^ concentration, arising from the strong interaction between F^−^ ions and binding sites in UiO-66 channels, is the primary reason for the observed high F^−^/Cl^−^ selectivity. Note that the enhanced F^−^ ion mobility at a high concentration (compared with a low concentration) in UiO-66 channels also contributes to the high selectivity. The high-performance synthetic F^−^ ion channels demonstrated here provide an attractive strategy for developing artificial fluoride ion channel membranes for applications such as efficient removal of toxic fluoride ions from water. Furthermore, MOFs with tailorable angstrom-sized pores have great potential as platforms for constructing other ion channels and separation membranes.

## Methods

### Materials

Zirconium (IV) chloride (ZrCl_4_), benzene-1,4-dicarboxylic acid or terephthalic acid (BDC), 2-aminoterephthalic acid (BDC-NH_2_), dimethylformamide (DMF), iodomethane (CH_3_I), potassium fluoride (KF), potassium chloride (KCl), potassium bromide (KBr), potassium iodide (KI), potassium nitrate (KNO_3_), potassium sulfate (K_2_SO_4_), sodium dodecyl diphenyloxide disulfonate, formic acid (HCOOH) and hydrochloric acid (HCl) were purchased from Sigma-Aldrich, Australia. Methanol, ethanol, potassium hydroxide (KOH) and sodium hydroxide (NaOH) were purchased from Merck, Australia. PET membranes were obtained from Hostaphan RN12 Hoechst, Germany.

### Nanochannels preparation

PET membranes (12 μm thick, with single or multiple ion tracks in the center) were simultaneously etched from one side with 6 M NaOH + 0.025% sodium dodecyl diphenyloxide disulfonate and the other side with 6 M NaOH etching solution at 60 °C to produce single or multiple bullet-shaped nanochannels. A Keithley 6487 picoammeter (Keithley Instruments, Cleveland) was employed to observe the changing current of the single-nanochannel membrane during etching. The etching process was terminated by adding a mixture of 1.0 M KCl and 1.0 M HCOOH aqueous solution, which neutralized the alkaline etching solution. The morphologies and diameters of the nanochannels were observed by SEM using multichannel membranes prepared under the same etching conditions as single channel membranes.

### Modification of single nanochannels with UiO-66-X MOFs

ZrCl_4_ (150 mg) and BDC (106 mg) or BDC-NH_2_ (120 mg) in DMF (25 mL) were ultrasonically dissolved in a glass bottle. The resulting clear solution was transferred into a Teflon-lined stainless-steel autoclave, in which the PET membrane with a single-nanochannel was secured vertically with a holder. Subsequently, the autoclave was placed in an oven and heated at 100 °C for 24 h. After cooling to room temperature, the as-prepared nanochannel membrane was washed with ethanol three times, followed by drying at room temperature overnight. Finally, the resulting single-channel membrane was immersed into CH_3_I methanol solution for 72 h for the quaternization process, followed by washing with methanol three times and drying at room temperature overnight.

### Current measurement

The current measurements were carried out with a Keithley 6487 picoammeter (Keithley Instruments, Cleveland), and a PET membrane with a single-channel was placed between two cells (Supplementary Fig. 4a,b). One platinum (Pt) electrode was placed in each cell and employed to apply a voltage across the nanochannel. The tip side of the nanochannel faced the anode, and the base side faced the cathode. A scanning voltage from −2 V to + 2 V with a period of 20 s was applied three times. For measurements of the anion selective transport properties of the nanochannel, different solutions including KF, KCl, KBr, KI, KNO_3_, and K_2_SO_4_ were added to both cells, respectively. The selectivity of F^−^ over Cl^−^, Br^−^, I^−^, NO_3_^−^, and SO_4_^2−^ was studied by recording the ionic current during the potential scan resulting from anions transport through the nanochanne.

### Molecular dynamics (MD) simulations

The simulation system is a supercell composed of 2 × 2 × 2-unit cells of UiO-66. We adopted the force field parameters from reference^51^ for UiO-66. The original TIP/3 P model was adopted for water, and the SHAKE algorithm was employed to permit a longer simulation and partially reduce the statistical error. The LJ parameters potentials of ions were taken from the DANG force field^52^. The Lorentz-Berthelot mixing rule was used to determine LJ potential parameters between different types of ions, water molecules and atoms of UiO-66. The detailed LJ potential parameters and partial charges are summarized in Supplementary Table 11. All simulations employed a 10 Å cut-off for van der Waals (vdW) interactions using modified LJ potential, short-range electrostatic forces, and long-range electrostatics that employed the particle-particle particle-mesh (PPPM) algorithm. All simulations were performed using the LAMMPS code.

### A slab model to estimate equilibrium F^−^ concentration

The concentration effect arising from the strong F–Zr interaction was examined via simulating the ion absorption at UiO-66 surface. A UiO-66 slab whose surfaces had several exposed half cavities was created (Supplementary Fig. 20a). This slab was connected to two reservoirs with electrolyte at a F^−^ concentration of 1.0 M. MD simulations were run under NVT ensemble conditions at 300 K. We checked the ion concentration in the reservoir during the MD simulations and gradually inserted more ions to maintain its concentration at 1.0 M. After thermal equilibrium for 15 ns, we calculated the average ion concentration distribution along the direction perpendicular to the slab surface. Supplementary Fig. 20b summarizes the F^−^ concentration inside the half-exposed cavities at the slab surface. The ion concentration in the cavities was then estimated as the number ratio of ions over water.

### Determination of water molecule number density within UiO-66 supercells

To determine the number of water molecules within UiO-66 supercells at thermo-equilibrium, we performed MD simulations on a UiO-66 slab connected with two reservoirs (or gaps) filled with water (Supplementary Fig. 10). Initially, the MOF supercells were filled with a prescribed number of ions (e.g., 1 ion in a 2 × 2 × 2 UiO-66 supercell or 96 ions in a 2 × 2 × 2 UiO-66 supercell) but no water molecules. We then ran MD simulations under an NVT ensemble to allow diffusion of water molecules diffused into the UiO-66 frameworks. Meanwhile, we gradually inserted more water molecules in the reservoirs to make the number density close to the value at ambient conditions. When the water number density in the reservoirs showed no changes for ~10 ns, we considered the systems to have reached the equilibrium conditions. Using the ratio of the number of ions to the number of water molecule number in the UiO-66 supercells, we were able to determine the ion concentration, i.e., 1 ion in the 2 × 2 × 2 supercell corresponding to 0.0457 M, and the 96 ions in the 2 × 2 × 2 supercell corresponding to 3.748 M.

### Applying external electric fields to determine ion mobility

NVT ensemble simulations were performed at 298 K. The time step was 1 fs, and an external electric field of 0.1 V Å^−1^ was applied. Using external electric fields to calculate ion mobility is a widely accepted practice in previous studies, including ion transport in bulk aqueous solutions^52,53^ and ion transport in nano-channels^54–59^. The electric field strength chosen in this study, i.e., 0.1 V Å^−1^, has been frequently used^57,59^ and is far below 1 V Å^−1^ (the upper bound of eletric field strength, beyond which the polarization of water molecules is notable)^60,61^. Results generated during the last ~ 35 ns were used to calculate the ion flow velocity and radial distribution function. The mobility values (*μ*) were calculated *via*:

1 $$\left\langle {\mu \left( t \right)} \right\rangle = \left\langle V \right\rangle /E$$

where *E* is the electric field strength (0.1 V Å^−1^), and *V* is the drift velocity. *V* is collected from the displacement of the ions in the presence of electric field. The ion displacement profiles (Supplementary Fig. 21) show that Cl^−^ ions (at 0.0457 M) and F^−^ ions (at 0.0457 M, or at concentration 3.748 M) are crossing pore windows repeatedly during mobility calculations. However, F^−^ ions (at 0.0457 M and using standard F–Zr LJ interaction strengths) crossed the pore window once during that ~35 ns. Thus, we performed an additional 4 MD simulations (for around 250 ns, collecting displacement values for 224 ns) for F^−^ ions at low concentration with the standard F–Zr LJ strength in order to obtained more accurate mobility results.

### Potential of mean force calculations

Using the collective variables (colvars) package developed by Fiorin and co-workers^62^, umbrella sampling calculations were performed within LAMMPS. The target ion was confined by a 40 kcal mol^−1^ Å^−2^ harmonic restraint acting along the reaction coordinate, with 30 umbrellas each having an approximate spacing of 0.3 Å. In each umbrella, the target ion was placed at the harmonic center. After 2 ns of NVT equilibrium at 300 K was run, we recorded the positions of the target ion along the reaction coordinate direction within each umbrella was recorded at 1000 fs intervals over a 4 ns NVT production period. The weighted histogram analysis method (WHAM) was utilized to combine the different umbrella simulations into a free energy curve^63^. Supplementary Fig. 8 depicts the 30 histograms of F^−^ and Cl^−^ ions at different positions along the straight line that connects the large cavity center to the small cavity center. Supplementary Fig. 9 shows the PMF results.

### Diffusion coefficients calculations

The self-diffusion coefficient of ions in UiO-66 channels is calculated by weighting the hopping rates out of the 8-coordinated cavity sites ($k_{A \to B}$) and 4-coordinated cavity sites ($k_{B \to A}$) by including the following weighting factors in the equation to modify the equilibrium probability of occupying cavity A (*P*_*A*_) and B (*P*_*B*_):^45^

2 $$D_S = \frac{1}{6}\lambda ^2\left( {8k_{A \to B}P_A + 4k_{B \to A}P_B} \right)$$

where *λ* (8.98 Å) is the hopping distance between adjacent cavities. Since the likelihood of occupying a site is directly proportional to the residence time in each site (the inverse of the hopping rate), the occupancy probabilities can be eliminated, yielding

3 $$D_S = \frac{1}{6}\lambda ^2\left( {8k_{A \to B}\left( {\frac{{k_{B \to A}}}{{2k_{A \to B} + k_{B \to A}}}} \right) + 4k_{B \to A}\left( {\frac{{2k_{A \to B}}}{{2k_{A \to B} + k_{B \to A}}}} \right)} \right)$$

The hopping rates $k_{A \to B}$ and $k_{B \to A}$ were calculated by applying 1-dimentional transition state theory (TST):

4 $$k_{A \to B} = k\sqrt {\frac{{k_BT}}{{2\pi m}}} \frac{{e^{ - \beta F\left( {q \ast } \right)}}}{{\int_{{\mathrm{Cage}}} {e^{ - \beta F\left( q \right)}dq} }}$$

where *m* is the mass of the adsorbate molecule and *k* is the Bennett-Chandler dynamic correction factor, which was assumed to be 1 in this work^64^. *F*(q^*^) is the free energy at the transition states. The denominator of equation (4) was evaluated by integrating over the points on the reaction coordinate associated with the respective cavity microstates. We estimated the self-diffusion coefficient to be 7.60 × 10^−10^ cm^2^ s^−1^ (which converts to a mobility value of 2.96 × 10^−8^ cm^2^ V^−1^∙s^−1^ using Einstein relation) and 9.40 × 10^−8^ cm^2^ s^−1^ (which converts to a mobility value of 3.66 × 10^−6^ cm^2^ V^−1^∙s^−1^) for F^−^ and Cl^−^ ions, respectively. These values agree reasonably well with our mobility data 1.5 × 10^−7^ cm^2^ V^−1^∙s^−1^ for F^−^ ions and 6 × 10^−6^ cm^2^ V^−1^∙s^−1^ for Cl^−^ ions, calculated in MD simulations using the external electric field methods. This further confirms the reliability of the electric field method.

### Powder X-ray diffraction (PXRD)

XRD patterns were recorded in the 2θ range of 2–50° at room temperature using a Miniflex 600 diffractometer (Rigaku, Japan) in transmission geometry, using Cu Kα radiation (15 mA and 40 kV) at a scan rate of 2° min^−1^ and a step size of 0.02°.

### Scanning electron microscopy (SEM)

SEM images were taken with a field-emission scanning electron microscope (FEI Magellan 400 FEG SEM) operating at 5 kV, 13 pA.

### X-ray photoelectron spectroscopy (XPS)

XPS spectra were recorded using a Kratos Axis UltraDLD instrument (KratosLtd., Telford, UK) that is equipped with a monochromated Alkα (1486 eV) source operating at 150 W (15 kV and 10 mA). The photoelectron take-off angle with respect to the normal surface in all measurements was 0°.

### Zeta potential

Zeta potentials of UiO-66-X crystals were measured and analyzed using a Zeta Sizer (Nano Series).

### Gas adsorption and desorption measurement

For gas adsorption/desorption isotherms, high-purity grade (99.999%) nitrogen was used throughout the experiments. Prior to gas adsorption/desorption measurement, UiO-66-X powders were activated at 140 °C for 24 h. Low pressure volumetric nitrogen adsorption/desorption isotherms up to 1 bar were measured by a Micromeritics 3 Flex gas sorption analyzer. BET surface areas and pore sizes were determined by measuring N_2_ adsorption/desorption isotherms at 77 K in a liquid nitrogen bath and calculated using the Micromeritics software. The DFT model was selected for characterizing of the pore size distribution.

### Ion conductivity

The ionic conductivity (*κ*) of a nanochannel can be defined as:

5 $$\kappa = G \cdot \frac{L}{S}$$

where *G* is the conductance of a nanochannel, *S* is its cross-sectional area, and *L* is its length. For MOF-filled nanochannels, *S* is the effective cross-sectional areas of MOF pores, which can be calculated from the pore volume of MOF crystals (see Supplementary Table 12 for further information).

For a bullet-shaped nanochannel, the radius profile *r(x)* can be described as:

6 $$r(x) = \frac{{r_b - r_t\exp ( - \frac{L}{h}) - (r_b - r_t)\exp ( - \frac{x}{h})}}{{1 - \exp ( - \frac{L}{h})}}$$

where *r*_*b*_ is the base radius, *r*_*t*_ is the tip radius, *L* is the length of a nanochannel, and *h* is a geometrical parameter characterizing the curvature of a nanochannel profile, designated as the curvature radius, which was observed by fitting the obtained experimental tip profiles^65,66^.

*L*/*S* of a bullet-shaped nanochannel is theoretically described as:

7 $$\frac{L}{S}{\mathrm{ = }}{\int}_0^L {\frac{1}{{\pi {\mathrm{r}}^2(x)}}} dx = {\int}_0^L {\frac{1}{{\pi \left[ {\frac{{r_b - r_t\exp ( - \frac{L}{h}) - (r_b - r_t)\exp ( - \frac{x}{h})}}{{1 - \exp ( - \frac{L}{h})}}} \right]^2}}} dx$$

At high electrolyte concentration (i.e., 1.0 M) and a pH value close to the isoelectric point of the surface (3.8), at which the electrical double layer can be neglected and the specific ion conductivity in a nanochannel is equal to that in bulk solution, the (*L*/*S*)_NC_ of a nanochannel can be calculated by:

8 $$\left( {\frac{L}{S}} \right)_{{\mathrm{NC}}} = \kappa \cdot \frac{U}{I}$$

where *k* is the ion conductivity of 1.0 M electrolyte in bulk solution, and *I* is the ion current measured at the applied voltage *U*. For MOF-filled nanochannels, (*L*/*S*)_MOF_ is calculated by:

9 $$\left( {\frac{L}{S}} \right)_{{\mathrm{MOF}}} = \left( {\frac{L}{S}} \right)_{{\mathrm{NC}}} \cdot \frac{1}{{v_{{\mathrm{MOF}}}d_{{\mathrm{calc}}}}}$$

where *v* is the pore volume of MOF crystals and *d*_calc_ is the calculated crystal density (see Supplementary Table 12 for further information).

## Supplementary information

Supplementary Information

## Data Availability

The data that support the findings of this study are available from the corresponding author upon reasonable request.
