# Supplementary material for: Fast and selective fluoride ion conduction in sub-1-nanometer metal-organic framework channels
Source: Nat Commun. 2019 Jun 11;10:2490. doi: 10.1038/s41467-019-10420-9 (PMC6560108; doi:10.1038/s41467-019-10420-9)
Supplement: Supplementary file 1 — Supplementary Information [file 41467_2019_10420_MOESM1_ESM.pdf]

Fast and selective fluoride ion conduction in sub-1-nanometer metal-organic framework channels

Li et al.

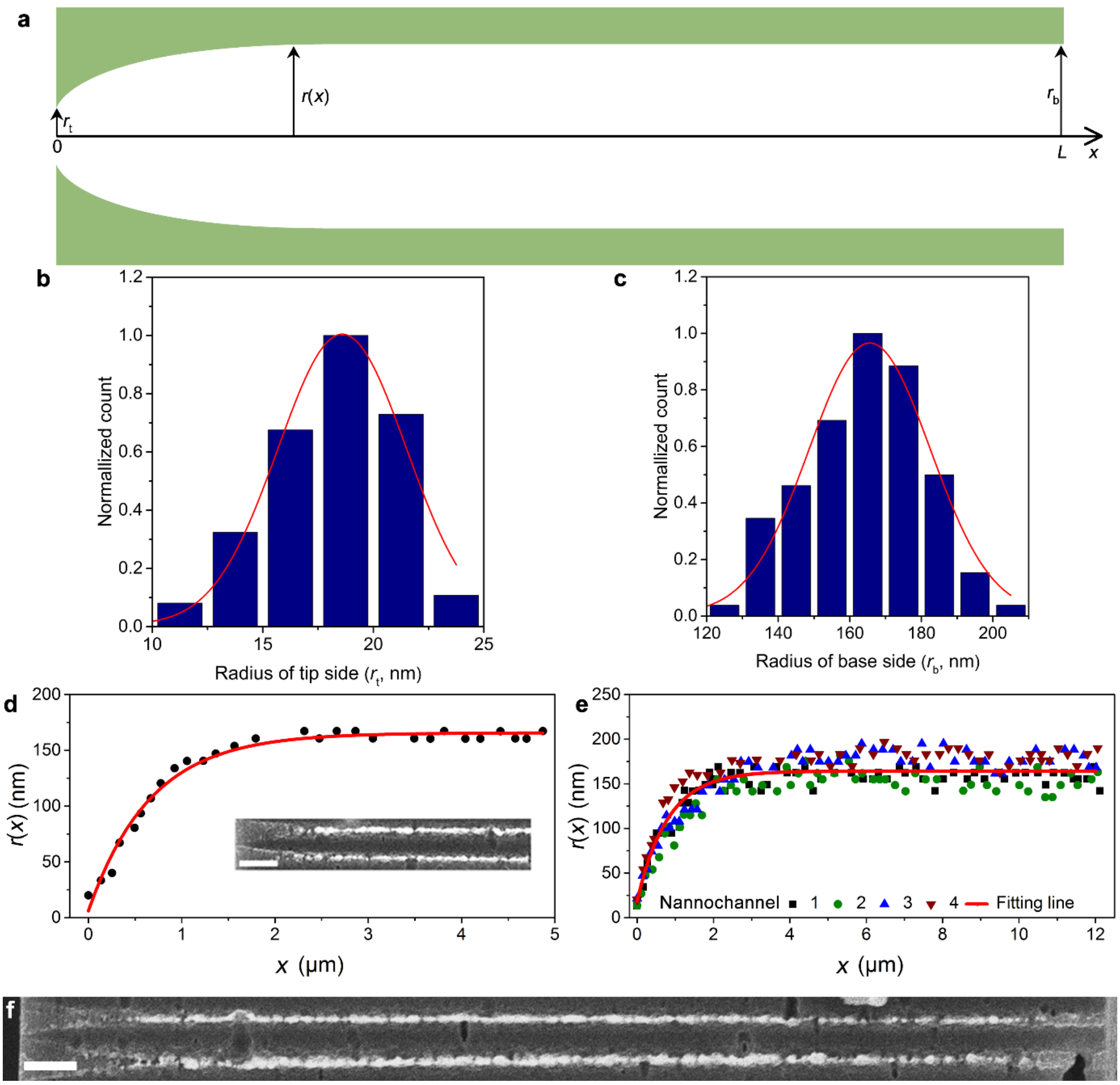

Supplementary Figure 1. Structural characterization of bullet-shaped nanochannels. **a**, Schematic of the cross section of a bullet-shaped nanochannel. **b**, Tip radius distribution, which has an average value of 18±3 nm. **c**, Base radius distribution, which has an average value of 164±18 nm. **d**, Experimental (points) and theoretical (red line) nanochannel tip radius profile. The inset is a SEM image of a nanochannel tip profile; the scale bar is 500 nm. **e**, Experimental (points labelled 1-4: four different nanochannel samples) and theoretical (red line calculated from equation (6), r_b = 164 nm, r_t = 18 nm, h = 800 nm, and L = 12 μm) nanochannel radius profiles. **f**, SEM image of the entire cross section of a bullet-shaped nanochannel, the scale bar is 500 nm.

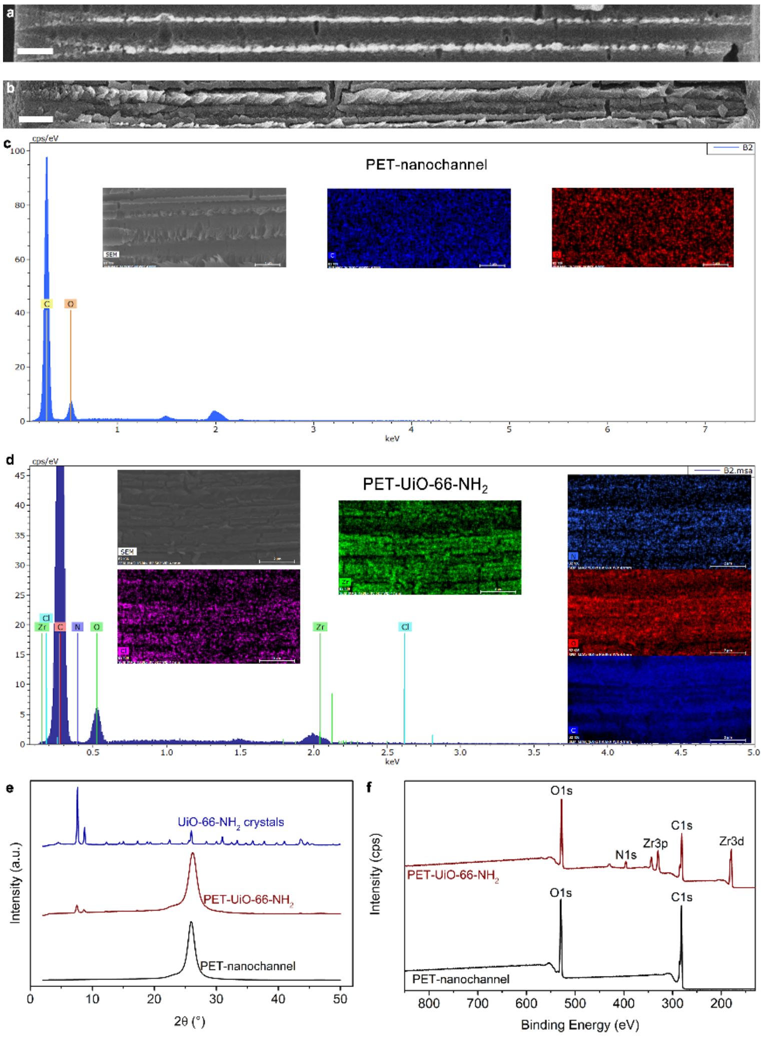

Supplementary Figure 2. EDX mapping of the cross sections and XRD and XPS spectra. **a**, **b**, SEM images of the entire cross section of the bullet-shaped nanochannel before and after UiO-66-NH_2 *in-situ* growth; the scale bars are 500 nm. **c**, EDX of a PET-nanochannel membrane (with a pore density of 10^8 cm^{-2}); the insets are an SEM cross-sectional image of a middle part of a PET multi-nanochannel membrane (left), and the EDX carbon (C) and oxygen (O) mappings (middle and right, respectively) along the cross section of the membrane; the scale bars are 1 µm. **d**, EDX of a PET-UiO-66-NH_2 membrane; the insets are an SEM cross-sectional image of a middle part of a PET-UiO-66-NH_2 multi-nanochannel membrane and EDX mappings of Cl (left), Zr (middle), and N, O, C (right) distributions; the scale bars are 2 µm. **e**, XRD patterns of the base surface of a PET-nanochannel, PET-UiO-66-NH_2 nanochannel, and UiO-66-NH_2 crystals. **f**, XPS spectra of the base surface of a PET-nanochannel and a PET-UiO-66-NH_2 nanochannel.

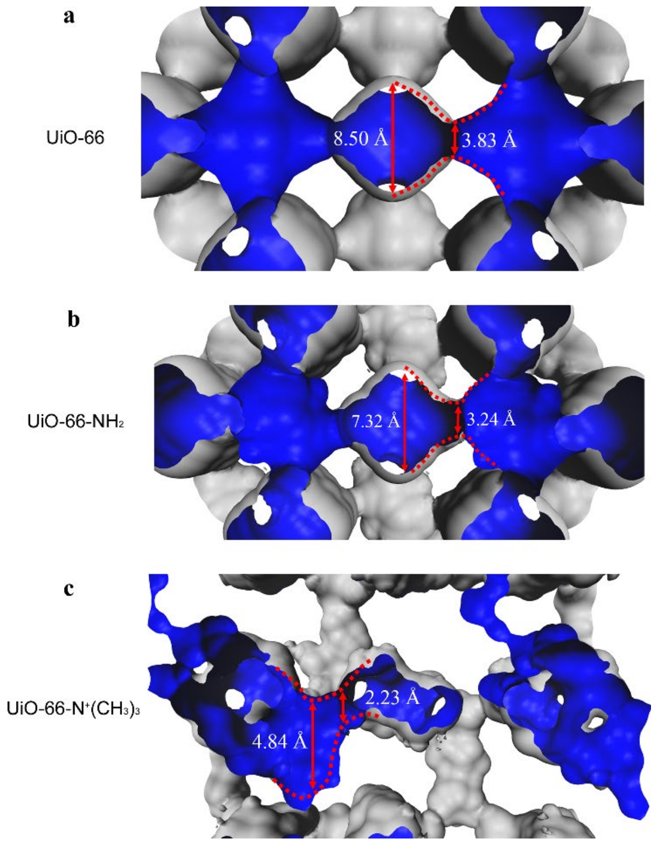

Supplementary Figure 3. UiO-66-X window and cavity sizes calculated via Zeo++[1]. **a**, Schematic of UiO-66 channels with a window diameter of 3.83 Å and a cavity diameter of 8.50 Å. **b**, Schematic of UiO-66-NH_2 channels with a window diameter of 3.24 Å and a cavity diameter of 7.32 Å. **c**, Schematic of UiO-66-$N^+(CH_3)_3$ channels with a window diameter of 2.23 Å and a cavity diameter of 4.84 Å.

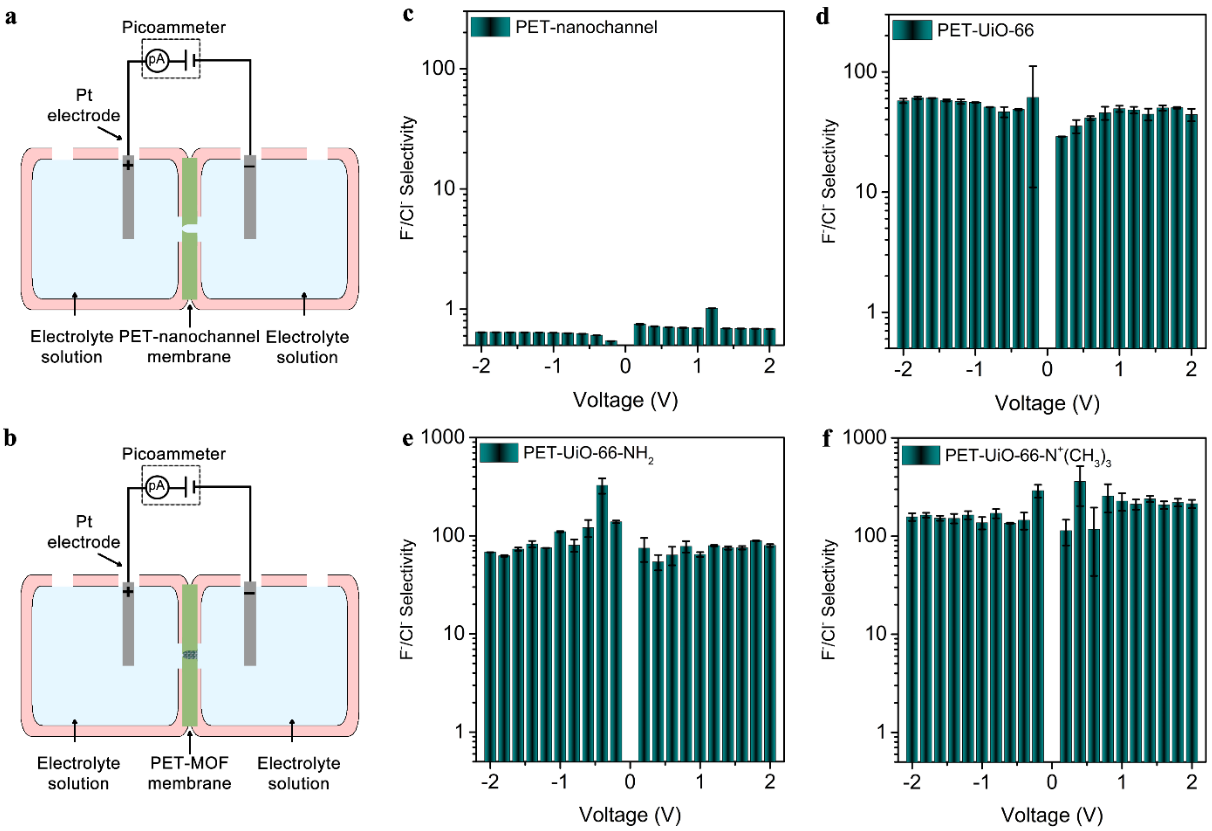

Supplementary Figure 4. F^-/Cl^- selectivity of PET and PET-MOF nanochannels. a, **b**, Schematics of experimental *I-V* curve measurement devices. **c**, F^-/Cl^- selectivity of a representative PET-nanochannel. **d**, F^-/Cl^- selectivity of a representative PET-UiO-66 nanochannel. **e**, F^-/Cl^- selectivity of a representative PET-UiO-66-NH_2 nanochannel. **f**, F^-/Cl^- selectivity of a representative PET-UiO-66-$N^+(CH_3)_3$ nanochannel. Error bars represent the standard deviation of three measurements of a sample.

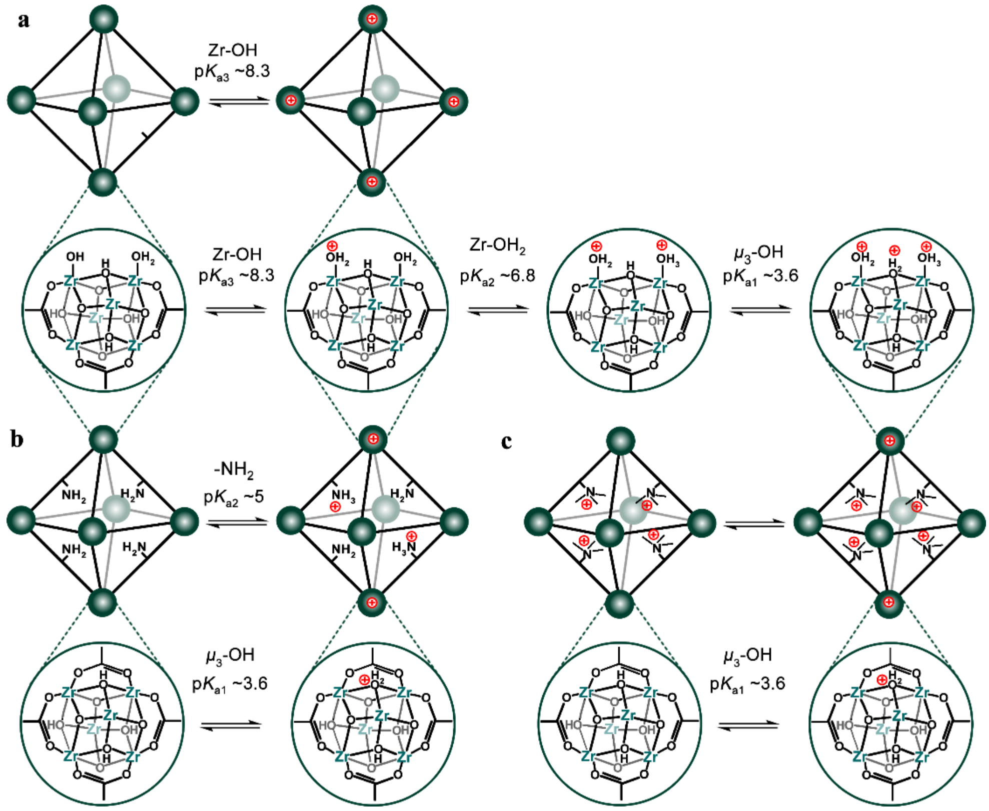

Supplementary Figure 5. pH-responsive property of UiO-66-X crystals. a, Schematics of pH-responsive properties of UiO-66. The pK_a of the μ_3–OH, Zr–OH_2 and Zr–OH groups on the Zr-nodes is about 3.52, 6.79 and 8.30 respectively[2]. **b**, pH responsive properties of UiO-66-NH_2. The pK_a of the NH_2 group on the ligand is about 5[3-5]. **c**, pH responsive properties of UiO-66-$N^+(CH_3)_3$.

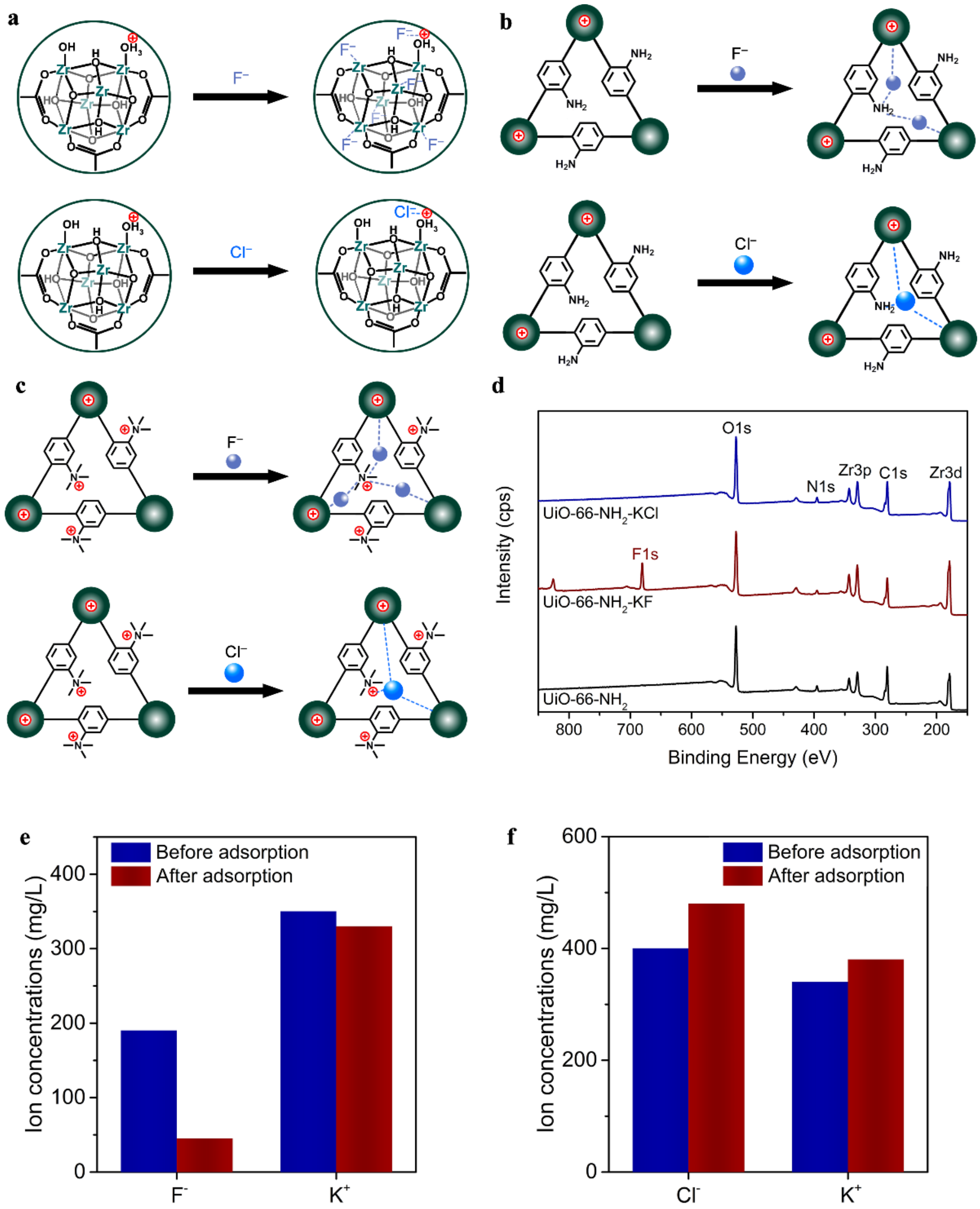

Supplementary Figure 6. Characterizations of UiO-66-X crystals before and after KF and KCl adsorption. **a**, Interaction of F^- and Cl^- with Zr sites, showing that F^- ions can specifically bind to Zr sites through specific Zr–F interaction (including replacing some –OH groups on Zr_6 nodes) and compensation by positively charged groups (i.e.–OH_2^+ and –OH_3^+ on Zr_6 nodes), while Cl^- ions only through compensation by positively charged groups. **b**, Hydrogen bonding interaction of F^- and Cl^- with NH_2 groups of UiO-66-NH_2. **c**, Electrostatic interaction of F^- and Cl^- with $N^+(CH_3)_3$ groups of UiO-66-$N^+(CH_3)_3$. **d**, XPS spectra of UiO-66-X (UiO-66-NH_2 as an example) before and after adsorption of F^- and Cl^- (see Supplementary Tables 3, 4 for elemental compositions). **e**, F^- and K^+ concentrations in bulk KF (0.01 M) solution before

and after adsorption measured by inductively coupled plasma optical emission spectrometry (ICP-OES) at ALS Environmental (Melbourne, Australia). **f**, Cl^- and K^+ concentrations in bulk KCl (0.01 M) solution before and after adsorption measured by ICP-OES. The adsorption capacity of UiO-66 (0.1 g) was calculated by $q = (V_0C_0 – VC)/m$, where V_0 is initial volume of electrolyte solution, V is the volume of electrolyte solution after adsorption, C_0 is initial ion concentration, C is the ion concentration of electrolyte solution after adsorption, and m is the quantity of UiO-66. The initial volumes of KF and KCl solutions were 25.0 mL, but the volume of KF solution decreased to 24.3 mL and the volume of KCl solution dropped to 22.2 mL after UiO-66 adsorption.

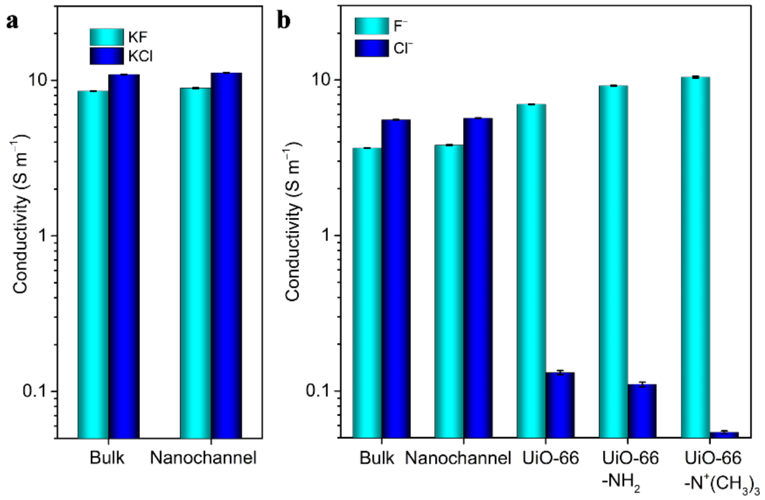

Supplementary Figure 7. Measured ionic conductivities of 1.0 M KF and KCl electrolytes. **a**, Ion conductivities of 1.0 M KF and KCl measured in bulk solution and PET-nanochannel. **b**, Ion conductivities of F^- and Cl^- measured in bulk solutions, PET-nanochannel, and PET-UiO-66-X nanochannels. Error bars represent the standard deviation of three measurements of a sample.

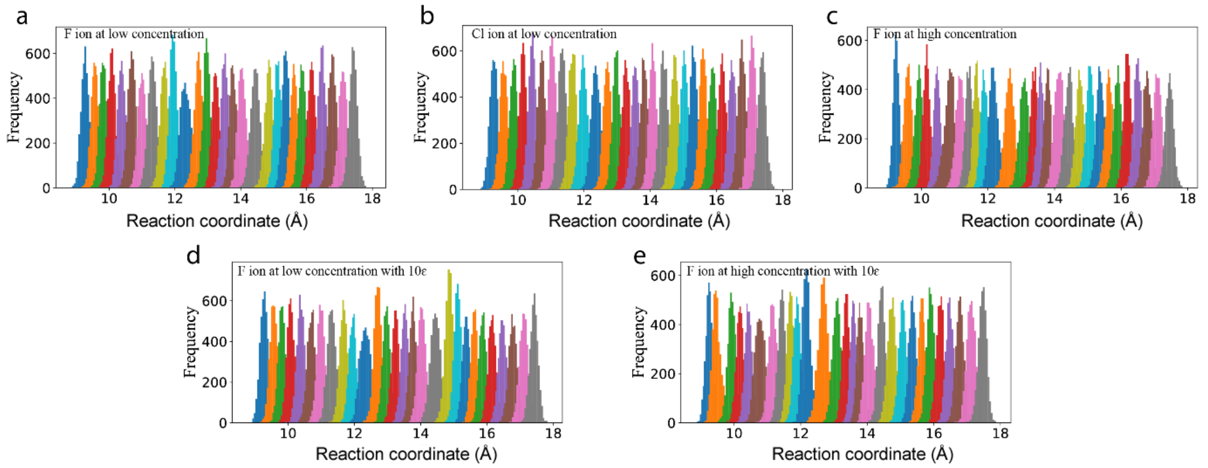

Supplementary Figure 8. Frequency histograms (umbrellas) for ion confined to 30 windows along the reaction coordinate by harmonic potentials at 300 K. **a**, For F^- ions at concentration 0.0457 M. **b**, Cl^- ions at concentration 0.0457 M. **c**, F^- ions at concentration 3.78 M. **d**, F^- ions at concentration 0.0457 M with 10ε. **e**, F^- ions at concentration 3.78 M with 10ε. We implemented umbrella sampling using the LAMMPS collective variable library[6]. The target ion was confined by a 40 kcal mol^{-1} Å^{-2} harmonic restraint acting along the reaction coordinate, with 30 umbrellas, each having an approximate spacing of 0.3 Å. In each umbrella, the target ion was placed at the harmonic centre. After 2 ns of NVT equilibrium at 300 K was run, the positions of target ions along the reaction coordinate direction within each umbrella were recorded at 1000 fs intervals over a 4 ns NVT production period.

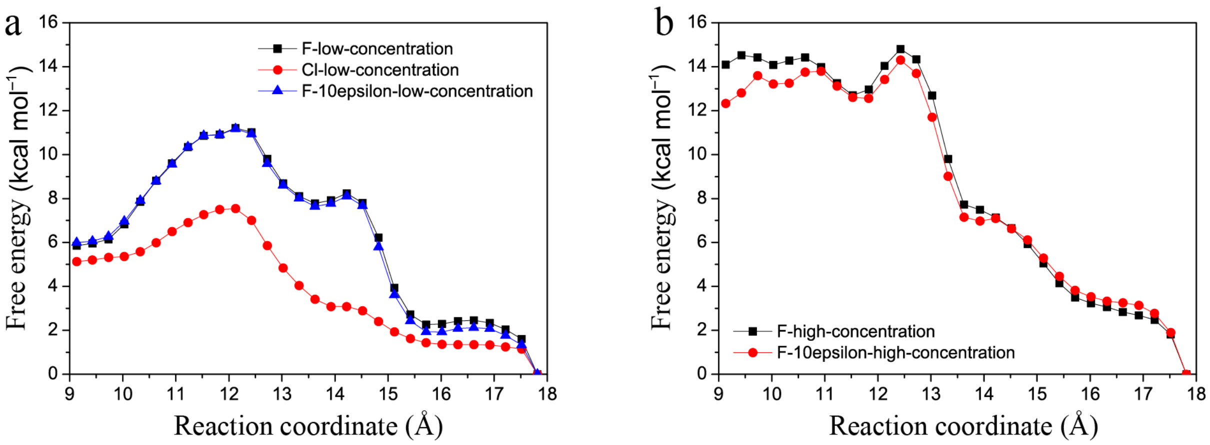

Supplementary Figure 9. Reconstructed free energy profiles from histograms with weighted histogram analysis method (WHAM) code[7]. **a**, Free energy profiles for ions at low concentration. **b**, Free energy profiles for ions at high concentration.

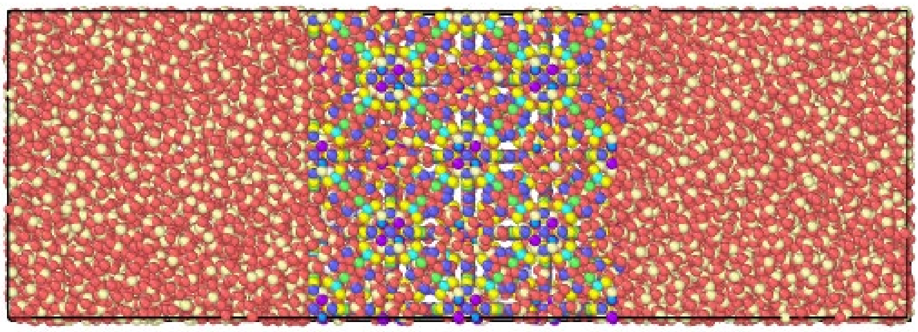

Supplementary Figure 10. A slab model to determine the thermodynamically equilibrated number of water molecule numbers within UiO-66 supercells.

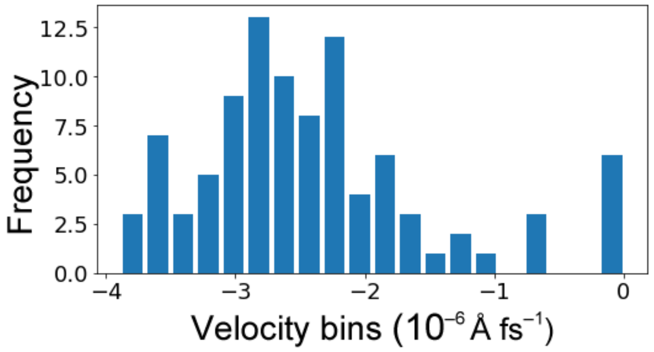

Supplementary Figure 11. Frequency histograms of averaged drift velocity for 96 F^- ions obtained from individual displacement-time relation during mobility calculations. This figure shows that most of the ions (around 93.75%) have drift velocities of the order of 10^{-6} Å fs^{-1}, and only 6 ions have smaller, but still non-zero, velocity values.

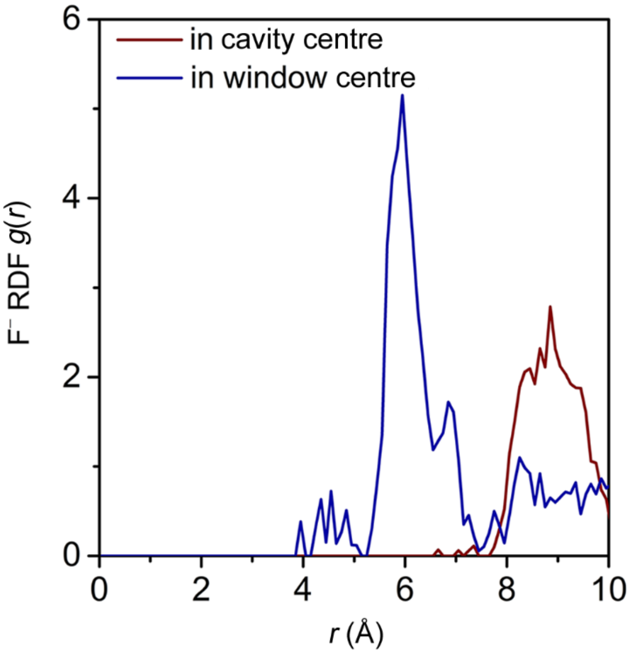

Supplementary Figure 12. Radial distribution function *g(r)* of F^- ions around a single F^- ion fixed in the large cavity centre and window centre of UiO-66.

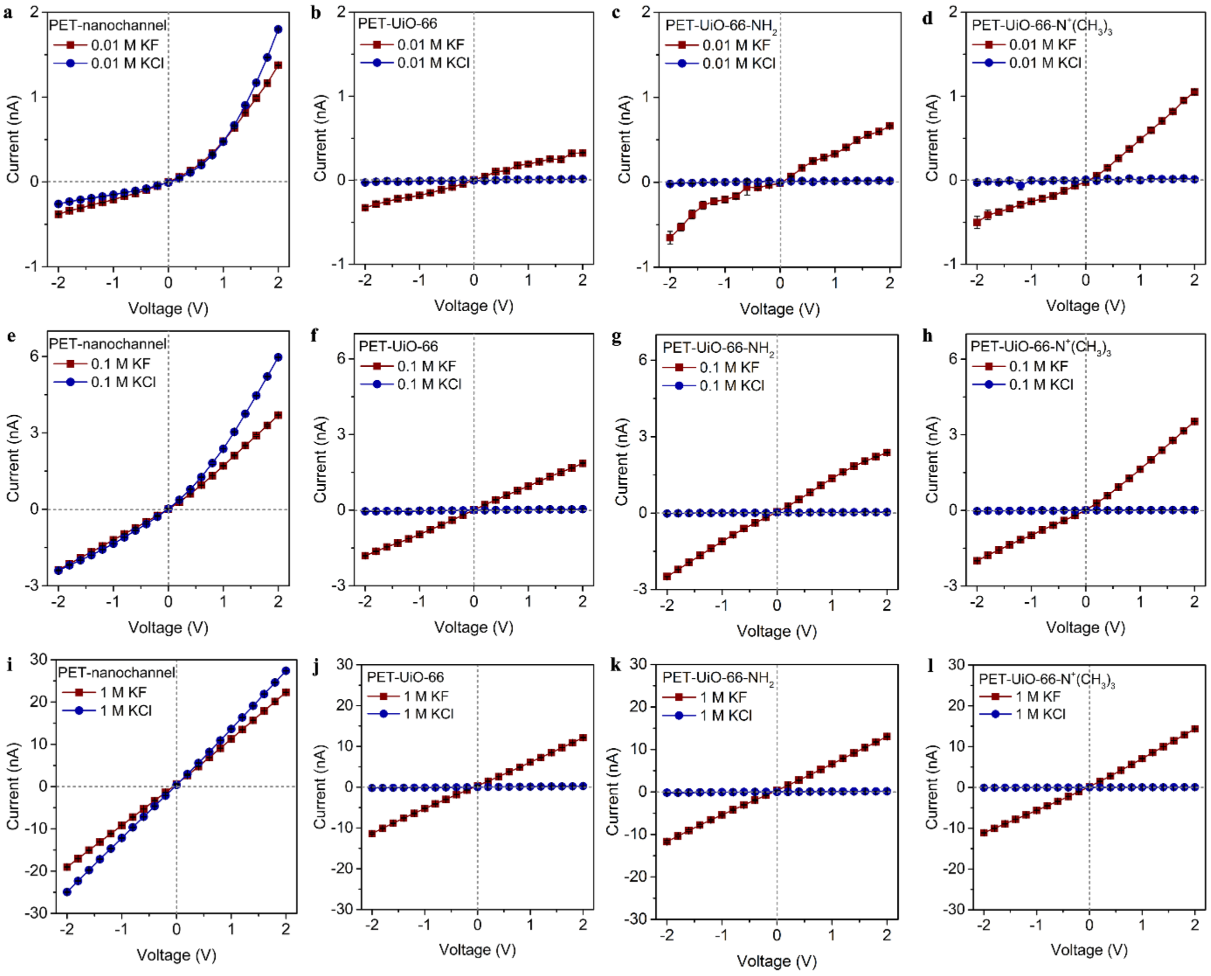

Supplementary Figure 13. *I-V* curves of PET and PET-UiO-66-X nanochannels as a function of external electrolyte concentrations. **a-d**, *I-V* curves of PET, PET-UiO-66, PET-UiO-66-NH_2, and PET-UiO-66-$N^+(CH_3)_3$ nanochannels measured in 0.01 M KCl and KF solutions, respectively. **e-h**, *I-V* curves of PET, PET-UiO-66, PET-UiO-66-NH_2, and PET-UiO-66-$N^+(CH_3)_3$ nanochannels measured in 0.1 M KCl and KF solutions, respectively. **i-l**, *I-V* curves of PET, PET-UiO-66, PET-UiO-66-NH_2, and PET-UiO-66-$N^+(CH_3)_3$ nanochannels measured in 1.0 M KCl and KF solutions, respectively. Error bars represent the standard deviation of three measurements of a sample.

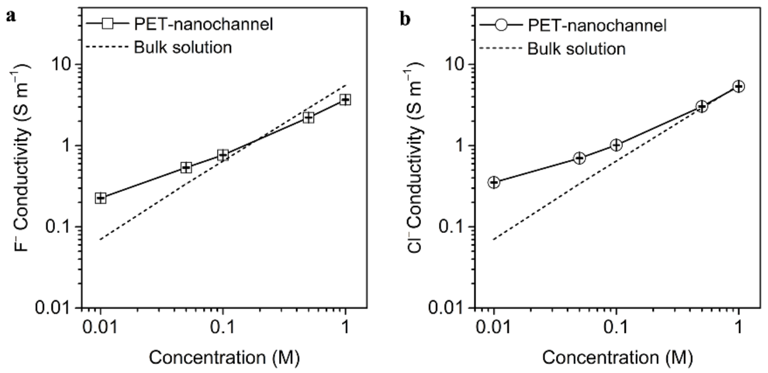

Supplementary Figure 14. F^{-} and Cl^{-} conductivities of a PET-nanochannel and bulk solution as a function of external electrolyte concentrations. **a**, F^{-} conductivities in the PET-nanochannel are higher than those in bulk solution at low concentrations (0.01–0.1 M) but close to the bulk solution values at high concentrations (0.5–1.0 M). **b**, Cl^{-} conductivities in the PET-nanochannel are higher than those in bulk solution at low concentrations (0.01-0.1 M) but close to the bulk solution values at high concentrations (0.5–1.0 M). This trend is because at low concentrations the electrical double layers in a PET-nanochannel contribute to the accumulation of ions in the nanochannel, leading to higher ion concentration in the PET-nanochannel than in bulk solution. Error bars represent the standard deviation of three measurements of a sample.

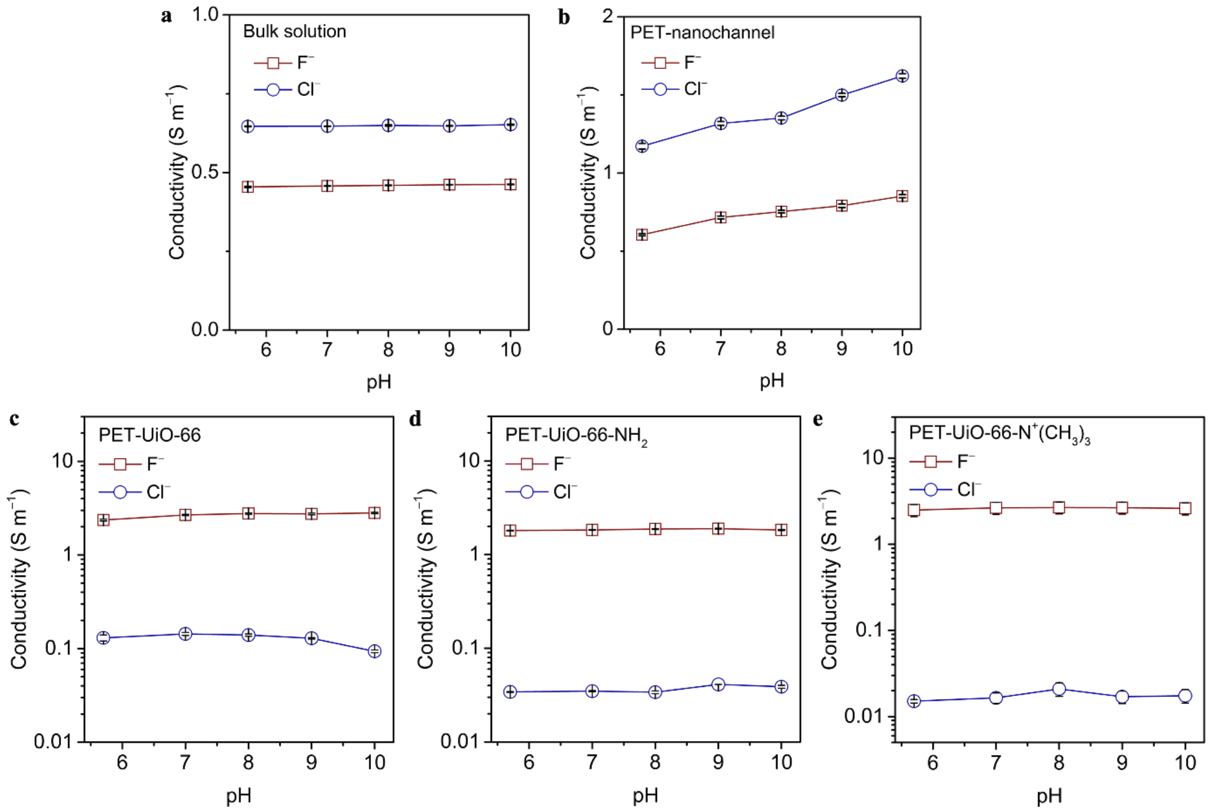

Supplementary Figure 15. pH responsive properties of PET and PET-UiO-66-X nanochannels. **a**, Ionic conductivities of 0.1 M F^- and Cl^- bulk solutions at different pH values. **b**, Effect of pH on ionic conductivities of 0.1 M F^- and Cl^- solutions in a PET-nanochannel. **c**, Effect of pH on ionic conductivities of 0.1 M F^- and Cl^- solutions in a PET-UiO-66 nanochannel. **d**, Effect of pH on ionic conductivities of 0.1 M F^- and Cl^- solutions in a PET-UiO-66-NH_2 nanochannel. **e**, Effect of pH on ionic conductivities of 0.1 M F^- and Cl^- solutions in a PET-UiO-66-$N^+(CH_3)_3$ nanochannel. Error bars represent the standard deviation of three measurements of a sample.

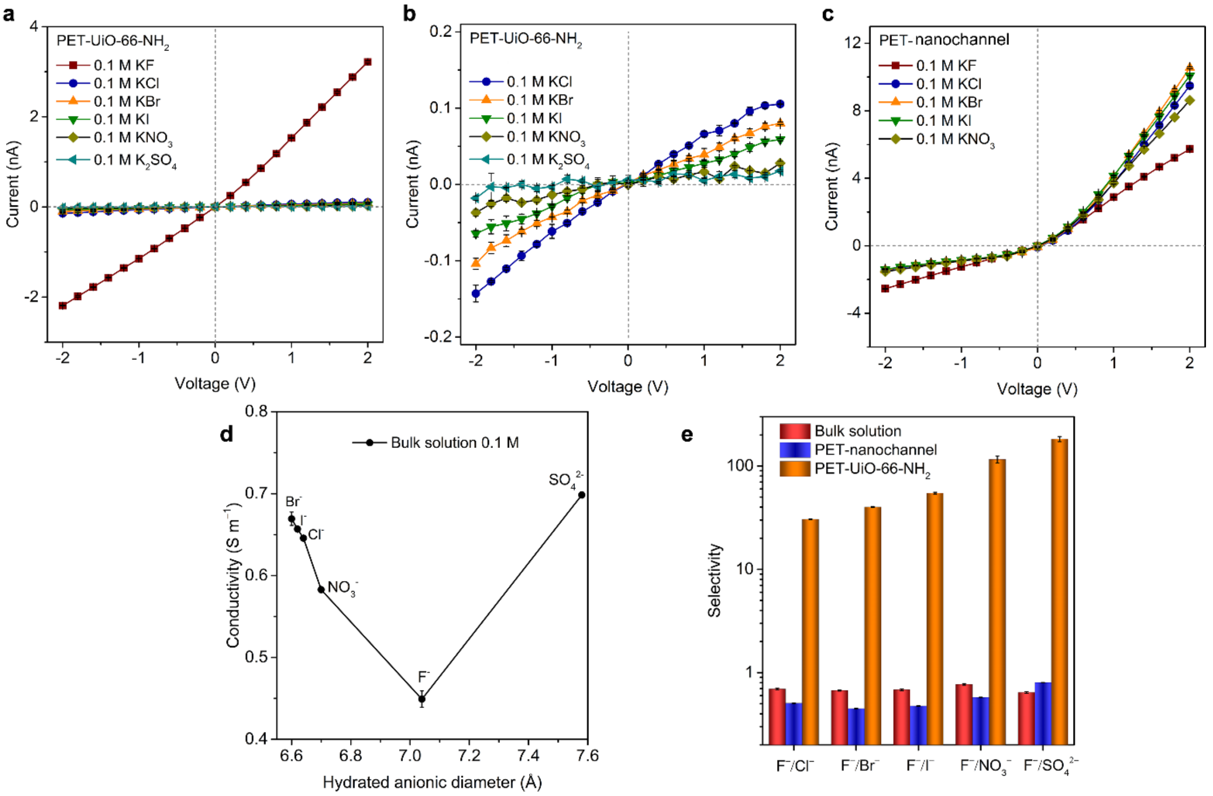

Supplementary Figure 16. Ionic conductivities of bulk solutions as well as a PET-nanochannel before and after growth of UiO-66-NH_2. a, *I-V* curves of a PET-UiO-66-NH_2 nanochannel measured in 0.1 M KCl, KBr, KI, KNO_3, K_2SO_4 and KF solutions. **b**, *I-V* curves of a PET-UiO-66-NH_2 nanochannel measured in 0.1 M KCl, KBr, KI, KNO_3 and K_2SO_4 solutions (expanded y axis from **a**). **c**, *I-V* curves of a PET-nanochannel measured in 0.1 M electrolyte solutions, including KF, KCl, KBr, KI and KNO_3. **d**, Conductivities of hydrated anions measured in 0.1 M (pH 5.7) bulk solutions. **e**, Anion selectivities (i.e., ratios of F^- to Cl^- conductivities) measured in bulk solution, PET-nanochannels, and PET-UiO-66-NH_2 nanochannels (3 repeated experiments were done for PET-UiO-66-NH_2 nanochannels and the error bars were calculated based on 3 measurement values of a nanochannel. See Supplementary Table 10 for individual results). Error bars represent the standard deviation of three measurements of a sample.

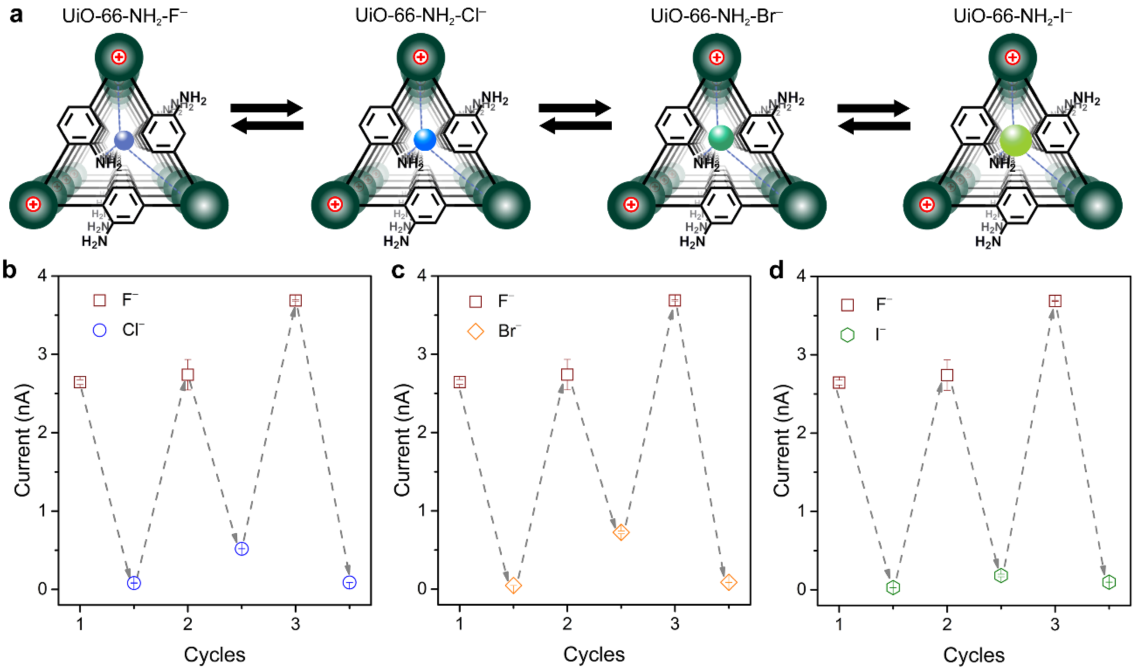

Supplementary Figure 17. Cyclic performance of PET-UiO-66-NH$_2$ nanochannels. a, Schematics of various anions (F^-, Cl^-, Br^- , and I^-) in UiO-66-NH$_2$ window pores. **b,** Current values for F^- and Cl^- during a 3 cycle test. **c,** Current values for F^- and Br^- during a 3 cycle test. **d,** Current values for F^- and I^- during a 3 cycle test. The PET-UiO-66-NH$_2$ nanochannel was immersed in methanol for 3 days after each cycle test. Error bars represent the standard deviation of three measurements of a sample.

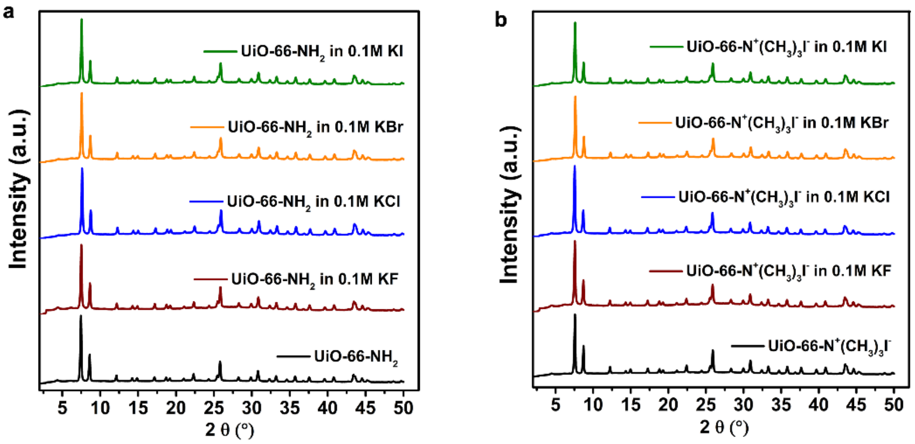

Supplementary Figure 18. Stability of UiO-66-NH_2 and UiO-66-$N^+(CH_3)_3$ crystals. a, XRD patterns of UiO-66-NH_2 before and after treatment in KF, KCl, KBr, and KI (0.1M, pH 5.7) solutions for 3 days. **b,** XRD patterns of UiO-66-$N^+(CH_3)_3$ before and after treatment in KF, KCl, KBr, and KI (0.1M, pH 5.7) solution for 3 days.

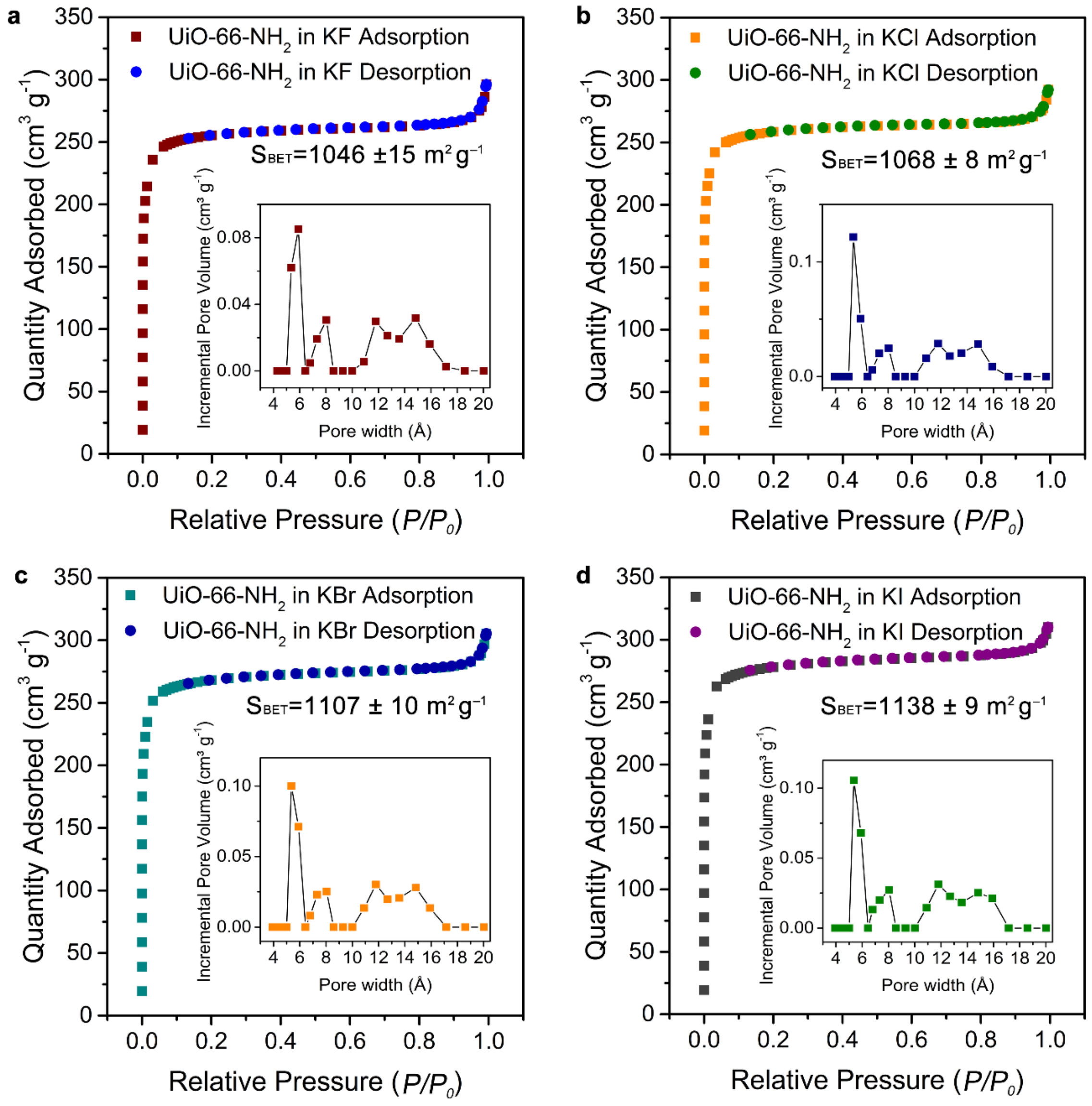

Supplementary Figure 19. Stability of UiO-66-NH_2 crystals. a, N_2 adsorption/desorption isotherm and pore size distribution of UiO-66-NH_2 after treatment in KF (0.1 M, pH 5.7) for 3 days. **b,** N_2 adsorption/desorption isotherm and pore size distribution of UiO-66-NH_2 after treatment in KCl (0.1 M, pH 5.7) for 3 days. **c,** N_2 adsorption/desorption isotherm and pore size distribution of UiO-66-NH_2 after treatment in KBr (0.1 M, pH 5.7) for 3 days. **d,** N_2 adsorption/desorption isotherm and pore size distribution of UiO-66-NH_2 after treatment in KI (0.1 M, pH 5.7) for 3 days.

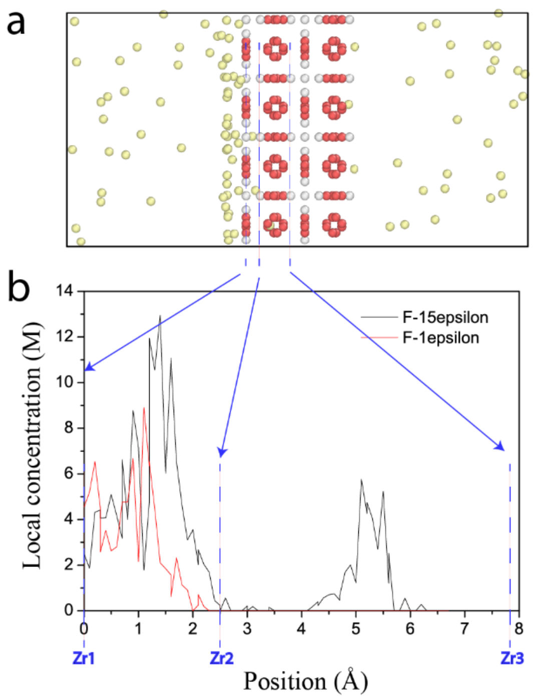

Supplementary Figure 20. MD simulations to estimate F^- concentration in a UiO-66 slab at different F–Zr vdW interaction strength. a, The simulation system includes a UiO-66 slab connected to two electrolyte reservoirs with ion concentration of 1.0 M. At the surfaces, there are some exposed half cavities, which makes equilibrium F^- adsorption in the cavities feasible in MD simulations. For clarity, only F atoms (yellow spheres), Zr atoms (white spheres), and C atoms (red spheres) are shown. During MD simulations, the ion numbers in the reservoirs were adjusted to keep the concentration close to 1.0 M. When the ion concentration in reservoirs showed no changes for 15 ns, we regarded it as having reached thermodynamic equilibrium. **b,** The line figure shows the F^- ion concentration profile along the direction perpendicular to UiO-66/solution interface.

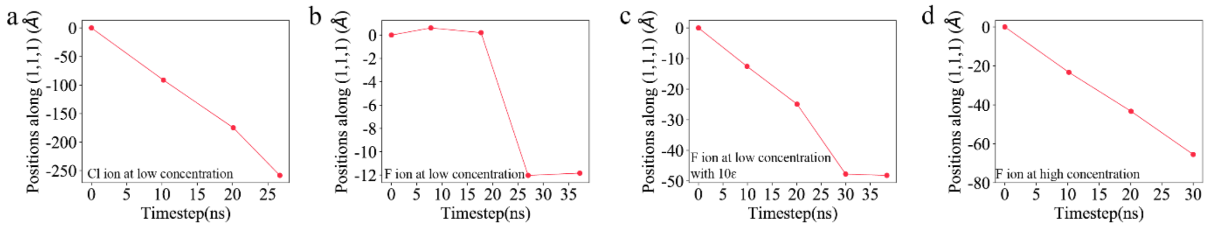

Supplementary Figure 21. Displacement profiles of ions as a function of time. **a**, Cl^- ions at concentration 0.0457 M. **b**, F^- ions at concentration 0.0457 M. **c**, F^- ions at concentration 0.0457 M with 10ε. **d**, F^- ions at concentration 3.78 M. The distance from the large cavity center to the adjacent small cavity center is 8.98 Å along the (1,1,1) direction. Ions crossed pore windows repeatedly in our MD simulations.

Supplementary Table 1. Ion hydrated and dehydrated diameters[8], and ionic mobilities[9], and hydration enthalpies[10].

Ion	Hydrated diameters (Å)	Dehydrated diameters (Å)	Mobilities (10^{-8} m^2 V^{-1} s^{-1})	Hydration Enthalpies (kJ mol^{-1})
K^+	6.62	2.66	7.62	−330
F^-	7.04	2.72	5.70	−520
Cl^-	6.64	3.62	7.91	−390
Br^-	6.60	3.90	8.13	−360
I^-	6.62	4.32	7.95	−320
NO_3^-	6.70	5.28	7.40	−320
SO_4^{2-}	7.58	5.80	8.27	−1080

Supplementary Table 2. Influence of aqueous anion solution composition on zeta potential of UiO-66-X MOFs.

MOF types	Zeta Potential /(mV)				
	H_2O	KF	KCl	KBr	KI
UiO-66	22.7±1.1	−10.5±0.4	3.7±0.2	6.1±0.8	23.0±0.3
UiO-66-NH_2	18.8±1.1	−11.5±0.6	5.6±1.2	7.8±0.1	19.3±0.7
UiO-66-$N^+(CH_3)_3$	35.1±4.4	−19.7±0.8	9.9±1.0	9.8±0.4	27.7±1.2

UiO-66-X crystals were dispersed in H_2O and in aqueous electrolyte solutions held at pH 5.7 containing 0.1 M KF, KCl, KBr, and KI. The MOF powder concentration in each solution was ~0.05 mg mL^{-1}, and readings were taken after 5 h. The values reported here represent averages and standard deviations from the results of three measurements.

Supplementary Table 3. XPS of UiO-66-NH_2 crystals before and after adsorption of various anions.

Samples	UiO-66-NH_2		UiO-66-NH_2+KF		UiO-66-NH_2+KCl		UiO-66-NH_2+KBr		UiO-66-NH_2+KI	
**Atomic%**	Mean	*Std*	Mean	*Std*	Mean	*Std*	Mean	*Std*	Mean	*Std*
**F 1s**	0.00	*0.00*	9.82	*0.09*	0.00	*0.00*	0.00	*0.00*	0.00	*0.00*
**O 1s**	31.99	*0.08*	32.62	*0.25*	34.01	*0.10*	34.19	*0.10*	35.48	*0.24*
**N 1s**	4.07	*0.02*	2.96	*0.18*	3.90	*0.19*	3.97	*0.11*	3.90	*0.15*
**C 1s**	56.42	*0.13*	44.25	*0.22*	53.75	*0.27*	53.82	*0.19*	52.53	*0.37*
**Zr 3d**	6.63	*0.04*	10.03	*0.04*	7.51	*0.01*	7.46	*0.18*	7.37	*0.06*
**Br 3d**	0.00	*0.00*	0.00	*0.00*	0.00	*0.00*	0.00	*0.00*	0.00	*0.00*
**Cl 2s**	0.64	*0.01*	0.00	*0.00*	0.37	*0.02*	0.24	*0.04*	0.19	*0.02*
**Si 2p**	0.27	*0.05*	0.34	*0.00*	0.47	*0.02*	0.34	*0.05*	0.55	*0.05*
**I 3d**	0.00	*0.00*	0.00	*0.00*	0.00	*0.00*	0.00	*0.00*	0.00	*0.00*

Supplementary Table 4. XPS of UiO-66 and UiO-66-$N^+(CH_3)_3$ I^- crystals before and after adsorption of various anions.

**Samples**	**UiO-66**		**UiO-66 + KF**		**UiO-66 + KCl**		**UiO-66-$N^+(CH_3)_3$ I^-**		**UiO-66-$N^+(CH_3)_3$ I^-+ KF**		**UiO-66-$N^+(CH_3)_3$ I^-+ KCl**	
**Atomic%**	Mean	*Std*	Mean	*Std*	Mean	*Std*	Mean	*Std*	Mean	*Std*	Mean	*Std*
**F 1s**	0.00	*0.00*	10.11	*0.23*	0.24	*0.02*	0.00	*0.00*	7.87	*0.05*	0.25	*0.01*
**O 1s**	21.68	*0.11*	32.24	*0.18*	30.64	*0.13*	29.20	*0.13*	33.63	*0.07*	33.96	*0.04*
**N 1s**	0.16	*0.01*	0.43	*0.03*	0.19	*0.04*	4.00	*0.24*	3.90	*0.15*	3.98	*0.01*
**C 1s**	73.20	*0.11*	47.16	*0.28*	62.22	*0.07*	59.44	*0.35*	44.97	*0.04*	53.67	*0.03*
**Zr 3d**	4.22	*0.01*	10.03	*0.04*	6.12	*0.13*	5.30	*0.01*	10.04	*0.06*	7.37	*0.01*
**Br 3d**	0.00	*0.00*	0.00	*0.00*	0.00	*0.00*	0.00	*0.00*	0.00	*0.00*	0.00	*0.00*
**Cl 2s**	0.38	*0.00*	0.06	*0.02*	0.09	*0.02*	0.45	*0.11*	0.00	*0.00*	0.10	*0.04*
**Si 2p**	0.30	*0.03*	0.33	*0.01*	0.39	*0.06*	1.44	*0.05*	0.45	*0.06*	0.42	*0.06*
**I 3d**	0.00	*0.00*	0.00	*0.00*	0.00	*0.00*	0.19	*0.02*	0.08	*0.01*	0.13	*0.01*
**S 2p**	0.07	*0.01*	0.18	*0.02*	0.13	*0.03*	0.00	*0.00*	0.11	*0.03*	0.13	*0.04*

Supplementary Table 5. XPS of UiO-66 crystals before and after adsorption of KF with different salt concentrations.

**Samples**	**UiO-66**		**UiO-66+ 0.01 M KF**		**UiO-66+ 0.1 M KF**		**UiO-66+ 0.5 M KF**		**UiO-66+ 1.0 M KF**	
**Atomic%**	Mean	*Std*	Mean	*Std*	Mean	*Std*	Mean	*Std*	Mean	*Std*
**F 1s**	0.00	*0.00*	1.47	*0.04*	10.11	*0.23*	30.14	*0.40*	32.73	*0.14*
**O 1s**	33.55	*0.02*	30.79	*0.32*	32.24	*0.18*	30.18	*0.23*	30.25	*0.23*
**C 1s**	59.56	*0.01*	61.54	*0.25*	47.16	*0.28*	18.84	*0.30*	14.62	*0.54*
**Zr 3d**	6.90	*0.02*	6.21	*0.02*	10.03	*0.04*	15.24	*0.01*	15.88	*0.11*
**K 2p**	0.00	*0.00*	0.00	*0.00*	0.00	*0.00*	5.61	*0.12*	6.26	*0.06*

Supplementary Table 6. F^-/Cl^-selectivity of biological fluoride ion channels.

**F^- channel type**	**F^-/Cl^- Selectivity**	**Reference**
CLC-pst N82M/T123V	9.2±1.2	11
CLC-eve M83N	11.2±1.2	11
WT	>10,000	12
N43D, pH 6.5	144.3±14.7	12
N43D, pH 7	62.0±7.0	12
F85I	65.0±6.7	12
F85I + Mb	10.0±0.8	12
F85I, pH 9	56.0±3.7	12

Supplementary Table 7. F^-/Cl^- selectivity for representative artificial fluoride channels and synthetic membranes.

Artificial fluoride channels/membranes		F^-/Cl^- Selectivity	Reference
Fluoride Transporter	Calix[4]pyrroles	1.8	13
Commercial Nanofiltration Membrane	NF 270	1.4	14
Anion Exchange Membrane (AEM)	NEOSEPTA AMX membrane	0.84	15

Supplementary Table 8. Ion mobility in bulk solution and in UiO-66 channels at 0.0457 M (in unit of 10^{-4} cm^2 V^{-1} s^{-1}) from MD simulations.

Ions	Bulk solution	UiO-66
Cl^-	5.23±0.46	0.058±0.013
F^-	4.22±0.40	0.0015±0.001
Ratio (F^-/Cl^-)	0.81	0.026

Supplementary Table 9. Influence of pH on zeta potential of UiO-66-X MOFs.

MOFs	Zeta Potential /(mV)				
	**pH=5.7**	**pH=7.0**	**pH=8.0**	**pH=9.0**	**pH=10.0**
UiO-66 (KF)	−20.9±1.2	−17.5±0.8	−16.0±0.5	−18.7±0.9	−17.8±1.0
UiO-66 (KCl)	11.4±1.1	17.3±0.9	12.4±0.7	13.3±0.6	10.4±2.0
UiO-66-NH_2 (KF)	−21.9±2.0	−19.2±1.2	−16.0±0.5	−21.4±0.4	−21.7±0.2
UiO-66-NH_2 (KCl)	5.6±1.2	3.9±0.2	4.2±0.2	4.8±0.3	7.0±2.9
UiO-66-$N^+(CH_3)_3$ (KF)	−25.4±3.8	−22.5±0.3	−18.6±0.6	−25.0±0.7	−26.3±2.1
UiO-66-$N^+(CH_3)_3$ (KCl)	20.2±0.8	19.0±0.9	21.5±1.3	21.5±0.6	23.7±8.3
UiO-66-NH_2 (Water)	/	17.2±2.1	12.9±0.7	19.2±0.4	22.1±0.5
	**pH=2.0**	**pH=3.0**	**pH=4.0**	**pH=5.0**	**pH=6.0**
UiO-66-NH_2 (Water)	53.5±1.6	44.9±2.9	38.3±2.6	21.3±0.6	17.1±0.8

UiO-66-X crystals were dispersed in aqueous electrolyte solutions with a pH value from 5.7 to 10 containing either 0.1 M KF or 0.1 M KCl respectively. UiO-66-NH_2 crystals were dispersed in aqueous solutions with pH values ranging from 2 to 10. The MOF powder concentration in each solution was ~0.5 mg mL^{-1}, and readings were taken after 5 h. The values reported here represent averages and standard deviations from the results of three measurements.

Supplementary Table 10. F^- selectivity over other anions for three representative PET-UiO-66-NH_2 nanochannels.

Sample	F^-/Cl^-	F^-/Br^-	F^-/I^-	F^-/NO_3^-	F^-/SO_4^{2-}
1	32.8	57.5	95.3	112.2	150.1
2	60.6	76.9	88.5	98.8	209.5
3	27.2	42.8	62.3	89.5	247.7
Average	40.2±17.9	59.1±17.1	82.0±17.4	100.2±11.4	202.4±49.2

Supplementary Table 11. Potential parameters and partial charges carried by each atom.

**Atomic types**	***σ* (Å)**	***ε* (kJ/mol)**	***q* (*e*)**
Zr	2.783	0.2887	+1.968
C1	3.473	0.3979	+0.630
C2	3.473	0.3979	–0.082
C3	3.473	0.3979	–0.065
O1	3.033	0.4004	–0.586
O3	3.033	0.4004	–0.992
H1	2.846	0.0636	+0.133
Na	2.876	0.5216	+1.000
F	3.143	0.6998	–1.000
Cl	3.785	0.5216	–1.000
O (H_2O)	3.151	0.6368	–0.834
H (H_2O)	0.000	0.0000	0.417

Supplementary Table 12. Crystallographic data for UiO-66-X (X= H, NH_2, and $N^+(CH_3)_3$).

Compound	UiO-66	UiO-66-NH_2	UiO-66-$N^+(CH_3)_3$
Formula	$Zr_6O_4(OH)_4(CO_2$-C_6H_4-$CO_2)_6$	$Zr_6O_4(OH)_4(CO_2$-C_6H_3-CO_2-$(NH_2))_6$	$Zr_6O_4(OH)_4(CO_2$-C_6H_3-CO_2-$(N^+(CH_3)_3))_6$
Pore volume (cm^3 g^{-1})	0.52256	0.40263	0.35637
Crystal density (g cm^{-3})	1.222	1.289	1.465

Note: The synthesized MOF UiO-66-$N^+(CH_3)_3$ has a counter-ion, which is I^-. But this counter-ion can be exchanged when testing various electrolyte solutions containing other anions (F^-, Cl^-, Br^-, and NO_3^-). Therefore, the structure (UiO-66-$N^+(CH_3)_3$) is used throughout the manuscript for brevity.

Supplementary References

1. Willems, T. F., Rycroft, C. H., Kazi, M., Meza, J. C. & Haranczyk, M. Algorithms and tools for high-throughput geometry-based analysis of crystalline porous materials. *Microporous Mesoporous Mater.* **149**, 134-141 (2012).
2. Klet, R. C., Liu, Y., Wang, T. C., Hupp, J. T. & Farha, O. K. Evaluation of bronsted acidity and proton topology in Zr- and Hf-based metal-organic frameworks using potentiometric acid-base titration. *J. Mater. Chem. A* **4**, 1479-1485 (2016).
3. Gross, K. C. & Seybold, P. G. Substituent effects on the physical properties and p*Ka* of aniline. *Int. J. Quantum Chem.* **80**, 1107-1115 (2000).
4. Liu, J., Cheng, L., Liu, B. & Dong, S. Covalent modification of a glassy carbon surface by 4-aminobenzoic acid and its application in fabrication of a polyoxometalates-consisting monolayer and multilayer films. *Langmuir* **16**, 7471-7476 (2000).
5. Hollingsworth, C. A., Seybold, P. G. & Hadad, C. M. Substituent effects on the electronic structure and p*Ka* of benzoic acid. *Int. J. Quantum Chem.* **90**, 1396-1403 (2002).
6. Fiorin, G., Klein, M. L. & Hénin, J. Using collective variables to drive molecular dynamics simulations. *Mol. Phys.* **111**, 3345-3362 (2013).
7. Grossfield, A. Wham: The weighted histogram analysis method. *Version* **2**, 06 (2012).
8. Nightingale, E. R. Phenomenological theory of ion solvation. Effective radii of hydrated ions. *J. Phys. Chem. A* **63**, 1381-1387 (1959).
9. Adamson, A. W. Chapter twelve - solutions of electrolytes. In: *A textbook of physical chemistry (second edition)*. (Academic Press, Cambridge 1979).
10. Smith, D. W. Ionic hydration enthalpies. *J. Chem. Educ.* **54**, 540 (1977).
11. Brammer, A. E., Stockbridge, R. B. & Miller, C. F^-/Cl^- selectivity in CLC^F-type F^-/H^+ antiporters. *J. Gen. Physiol.* **144**, 129-136 (2014).
12. Stockbridge, R. B. *et al.* Crystal structures of a double-barrelled fluoride ion channel. *Nature* **525**, 548-551 (2015).
13. Clarke, H. J. *et al.* Transmembrane fluoride transport: Direct measurement and selectivity studies. *J. Am. Chem. Soc.* **138**, 16515-16522 (2016).
14. Hong, S. U., Malaisamy, R. & Bruening, M. L. Separation of fluoride from other monovalent anions using multilayer polyelectrolyte nanofiltration membranes. *Langmuir* **23**, 1716-1722 (2007).
15. Vaselbehagh, M., Karkhanechi, H., Takagi, R. & Matsuyama, H. Surface modification of an anion exchange membrane to improve the selectivity for monovalent anions in electrodialysis – experimental verification of theoretical predictions. *J. Membr. Sci.* **490**, 301-310 (2015).
